# The Hippo-YAP signaling pathway drives CD24-mediated immune evasion in esophageal squamous cell carcinoma via macrophage phagocytosis

**DOI:** 10.1038/s41388-023-02923-z

**Published:** 2024-01-02

**Authors:** Xiaofeng Zhou, Ziyi Yan, Jinghan Hou, Lichen Zhang, Zhen Chen, Can Gao, Nor Hazwani Ahmad, Mingzhou Guo, Weilong Wang, Tao Han, Tingmin Chang, Xiaohong Kang, Lidong Wang, Yinming Liang, Xiumin Li

**Affiliations:** 1 Henan Key Laboratory of Tumor Molecular Therapy Medicine, The Third Affiliated Hospital of Xinxiang Medical University, Xinxiang, 453003 Henan Province PR China; 2 Xinxiang Key Laboratory for Molecular Therapy of Cancer, Xinxiang Medical University, Xinxiang, 453003 Henan Province PR China; 3 Department of Gastroenterology, The Third Affiliated Hospital of Xinxiang Medical University, Xinxiang, 453003 Henan Province PR China; 4 Henan Key Laboratory of immunology and targeted therapy, School of Laboratory Medicine, Henan Collaborative Innovation Center of Molecular Diagnosis and Laboratory Medicine, Xinxiang Medical University, Xinxiang, 453003 Henan Province PR China; 5 Department of Biomedical Science Advanced Medical and Science Institute, Universiti Sains Malaysia, Bertam 13200 Kepala Batas, Pulau Pinang, Malaysia; 6 Department of Gastroenterology & Hepatology, Chinese PLA General Hospital, #28 Fuxing Road, Beijing, 100853 PR China; 7 Department of Gastroenterology, The First Affiliated Hospital of Xinxiang Medical University, Xinxiang, 453003 Henan Province PR China; 8 Department of Oncology, The First Affiliated Hospital of Xinxiang Medical University, Xinxiang, 453003 Henan Province PR China; 9 State Key Laboratory of Esophageal Cancer Prevention & Treatment and Henan Key Laboratory for Esophageal Cancer Research of The First Affiliated Hospital, Zhengzhou University, Zhengzhou, 450052 China

**Keywords:** Oncogenes, Checkpoint signalling

## Abstract

Esophageal squamous cell carcinoma (ESCC) is one of the most lethal malignancies in the world with poor prognosis. Despite the promising applications of immunotherapy, the objective response rate is still unsatisfactory. We have previously shown that Hippo/YAP signaling acts as a powerful tumor promoter in ESCC. However, whether Hippo/YAP signaling is involved in tumor immune escape in ESCC remains largely unknown. Here, we show that YAP directly activates transcription of the “don’t eat me” signal CD24, and plays a crucial role in driving tumor cells to avoid phagocytosis by macrophages. Mechanistically, YAP regulates CD24 expression by interacting with TEAD and binding the CD24 promoter to initiate transcription, which facilitates tumor cell escape from macrophage-mediated immune attack. Our animal model data and clinical data show that YAP combined with CD24 in tumor microenvironment redefines the impact of TAMs on the prognosis of ESCC patients which will provide a valuable basis for precision medicine. Moreover, treatment with YAP inhibitor altered the distribution of macrophages and suppressed tumorigenesis and progression of ESCC in vivo. Together, our study provides a novel link between Hippo/YAP signaling and macrophage-mediated immune escape, which suggests that the Hippo-YAP-CD24 axis may act as a promising target to improve the prognosis of ESCC patients.

**Figure:**
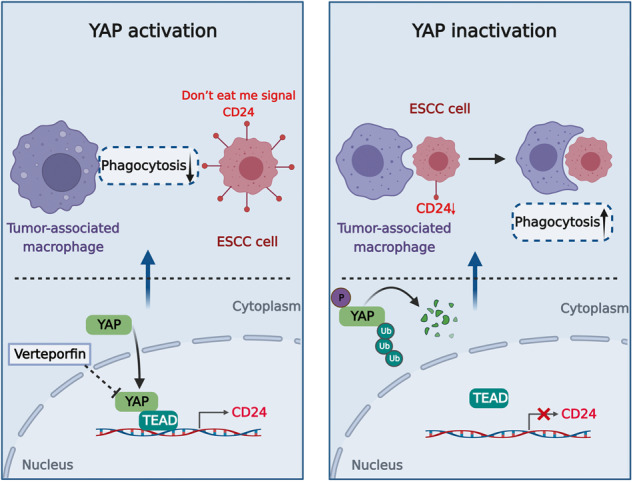
A proposed model for the regulatory mechanism of Hippo-YAP-CD24-signaling axis in the tumor-associated macrophages mediated immune escape.

A proposed model for the regulatory mechanism of Hippo-YAP-CD24-signaling axis in the tumor-associated macrophages mediated immune escape.

## Introduction

Esophageal cancer is one of the most aggressive malignancies and has been ranked as the sixth leading cause of cancer-related death in the world. According to the latest data from the International Agency for Research on Cancer (IARC), approximately 604,000 new esophageal cancer cases and 544,000 deaths occurred in 2020 [1]. More than half of all esophageal cancer patients were diagnosed in China, among which esophageal squamous cell carcinoma (ESCC) is the major histological type [2, 3]. As most patients were diagnosed at advanced stages and chemo-radiotherapy and immunotherapy proved ineffective, the five-year survival remains below 20% [4]. It is necessary to better understand the mechanism of ESCC progression for further development of novel targeting therapeutics. Immunotherapy with checkpoint inhibitors have been demonstrated to clinically benefit patients with different cancers [5]. Anti-Cytotoxic T lymphocyte-associated protein 4 (CTLA-4) and programmed cell death 1 (PD-1) antibodies are two of the most important checkpoint inhibitors [6]. However, for many tumor types, single drug checkpoint inhibitors are ineffective. PD-1/PD-L1 antibody treatment alone had almost no effect on pancreatic ductal adenocarcinoma (PDAC) [7]. For microsatellite instability-high (MSI-H) noncolorectal cancer patients, the response rate is below 20% [8]. Even in immune-responsive tumor types, most patients have shown resistance to checkpoint inhibitors after a period of therapy [6]. The development of predictors of response to immunotherapy and rational combinational therapeutics is urgently needed.

Tumor-associated macrophages (TAMs) are one of the most abundant immune-related stromal cells in the tumor microenvironment (TME). TAMs function like a double-edged sword in tumor development. TAMs foster angiogenesis and facilitate tumor growth and metastasis [9]. Meanwhile, enhancing TAM-mediated tumor cell phagocytic activity is a promising therapeutic strategy [10]. Previous studies have reported that the phagocytic activity of TAMs was sufficient to eradicate tumor cells, but the cancer cells were capable of evading attack by macrophages through the so called “don’t eat me” signals, including the overexpression of CD47, PD-L1, β2-microglobulin (B2M), and CD24 [11–15]. Monoclonal antibodies that block the interaction of “don’t eat me” signals with their macrophage-expressed receptors have demonstrated therapeutic potential in several cancers [16, 17]. Recently, CD24 has been reported as a novel innate immune checkpoint in cancer. By binding the inhibitory receptor sialic acid-binding Ig-like lectin 10 (Siglec-10) on TAMs, CD24 serves as an antiphagocytic or “don’t eat me” signal which helps tumor cells avoid phagocytosis by macrophages.

YAP is a transcriptional coactivator that functions as a core effector of the Hippo pathway and plays a key role in regulating multiple biological functions, including organ development, regeneration, and cancer biology [18–20]. YAP has been considered to be an oncogene in a number of cancer types, and its dysregulation often leads to tumor aggressiveness and metastasis [21–24]. We previously showed that YAP acted as a powerful tumor promoter in ESCC [25]. Although most previous research on YAP has focused on the biology of cancer cells themselves, growing evidence indicates that the TME plays a critical role in cancer development [26, 27]. Recently, emerging evidence suggests that YAP has an immunomodulatory effect in malignant tumors [28] and increasing YAP activity promotes PD-L1 expression and evasion of T-cell immune responses [29, 30]. These results indicate an interesting link between YAP activation and immune evasion processes, but the direct effect of YAP activation on the phagocytosis of tumor cells by macrophages is largely unknown.

In this study, unexpectedly, we found YAP directly activated transcription of the “don’t eat me” signal CD24, and played an important role in driving tumor cells to avoid phagocytosis by macrophages. Depletion of YAP downregulated CD24 expression and markedly promoted the phagocytosis of tumor cells by macrophages. In contrast, activation of YAP induced CD24 expression and inhibited macrophage phagocytosis, which was reversed by blocking CD24. Mechanistically, YAP regulates CD24 expression by interacting with TEAD and binding the CD24 promoter to initiate transcription, which facilitates tumor cells to escape from the attack of macrophages. Moreover, our clinical data suggested that CD24 and YAP were important evaluation factors affecting the prognosis of TAMs in ESCC. Taken together, our results uncovered a novel mechanism of macrophage-mediated tumor immune escape by YAP through regulating the “don’t eat me” signal CD24. More importantly, our findings indicated that targeting the YAP-CD24 axis has a dual role in inhibiting tumor growth and promoting macrophage phagocytosis of tumor cells which could be a promising strategy to improve prognosis of ESCC patients.

## Results

### YAP depletion significantly suppressed cell proliferation, migration and invasion in ESCC cells

**Fig. 1:**
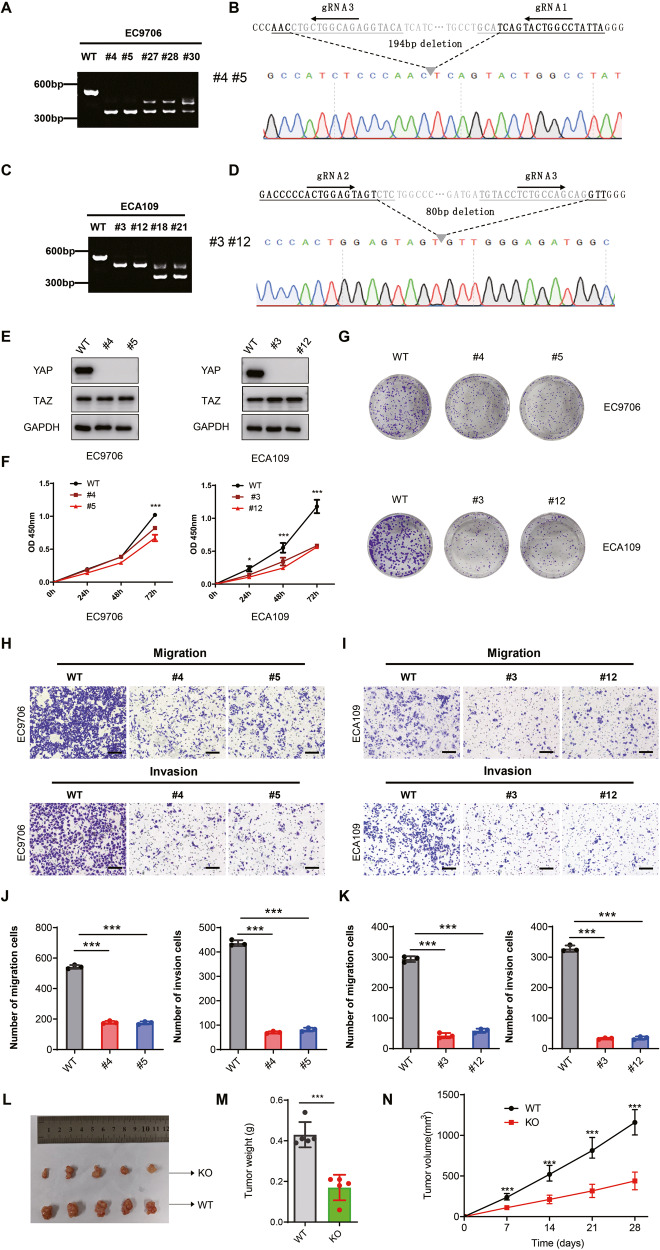
YAP knockout inhibited the proliferation, migration and invasion of human esophageal squamous cells. **A** PCR product from genomic DNA of selected clone4(#4) and clone5(#5) showing homozygous deletion of YAP in EC9706. **B** Sanger sequencing of clone4 and clone5 showing deletion of part of the YAP coding sequence. **C** PCR product from genomic DNA of selected clone3(#3) and clone12(#12) showing homozygous deletion of YAP in ECA109. **D** Sanger sequencing of clone3 and clone12 showing deletion of part of the YAP coding sequence. **E** Immunoblotting analysis showing CRISPR-mediated deletion of YAP in ESCC cell lines. **F** Cell viability was determined at indicated time points using CCK8 assay. Data are shown as mean ± s.e.m. ***P < 0.001. **G** Clone formation efficiency of WT and YAP KO cells. **H**, **I** YAP knockout inhibited the migration and invasion of different human esophageal squamous cells as determined by Transwell assays. Scale bars, 100 μm. **J**, **K** Quantification of migration and invasion cells. ***P < 0.001. L–N Representative tumor size, quantification of tumor weight and tumor growth curves from xenograft mouse models.

To investigate the effect of YAP loss of function on human ESCC cells, we employed the CRISPR-Cas9 technique to completely knockout YAP (YAP KO) expression in EC9706 and ECA109, which had a higher expression of YAP among 8 ESCC cell lines (Supplementary Fig. 1A). Three unique guide RNAs (gRNA) were designed to target exon2 of the YAP gene. After sorting, we picked approximately 30 clones and analyzed them via PCR for YAP KO using a primer pair flanking the gRNA target sites. The PCR results for these clones were analyzed, and it was found that EC9706 and ECA109 cells each had two homozygous clones (Fig. 1A and Fig. 1C). Sanger sequencing confirmed the homozygous deletion of 194 bp in EC9706 cells from clone 4 and clone 5 (Fig. 1B) and homozygous deletion of 80 bp in ECA109 cells from clone3 and clone 21(Fig. 1D). Additionally, after cell expansion, we simultaneously detected the expression of YAP and its paralog TAZ. We found that in some clones, weak bands of YAP expression were still observed on the Western blot results (#28 and #30 for EC9706, and #18 for ECA109), while other clones exhibited compensatory upregulation of TAZ expression (#27 for EC9706 and #21 for ECA109). Ultimately, we selected clones that had complete YAP knockout without affecting TAZ expression for further experiments (Fig. 1E and Supplementary Fig. 1C). YAP is a key component of the Hippo signaling pathway and plays a critical role in the development and progression of cancer. Therefore, we investigated the effects of YAP on the proliferation, migration, and invasion of ESCC cells. The CCK8 assay showed that YAP depletion significantly decreased cell proliferation (Fig. 1F) and clone formation results showed that YAP depletion dramatically inhibited the clone formation capacity in EC9706 and ECA109 cells (Fig. 1G). Furthermore, knockout of YAP in EC9706 and ECA109 cells significantly reduced their ability to migrate and invade (Fig. 1H, I). The statistical results are shown in the Supplementary Fig. 1B and Fig. 1J, K. Finally, in the tumor formation assay, depletion of YAP significantly inhibited xenograft tumor formation in immunodeficient mice (Fig. 1L–N).

### YAP depletion downregulates CD24 expression in ESCC cells

**Fig. 2:**
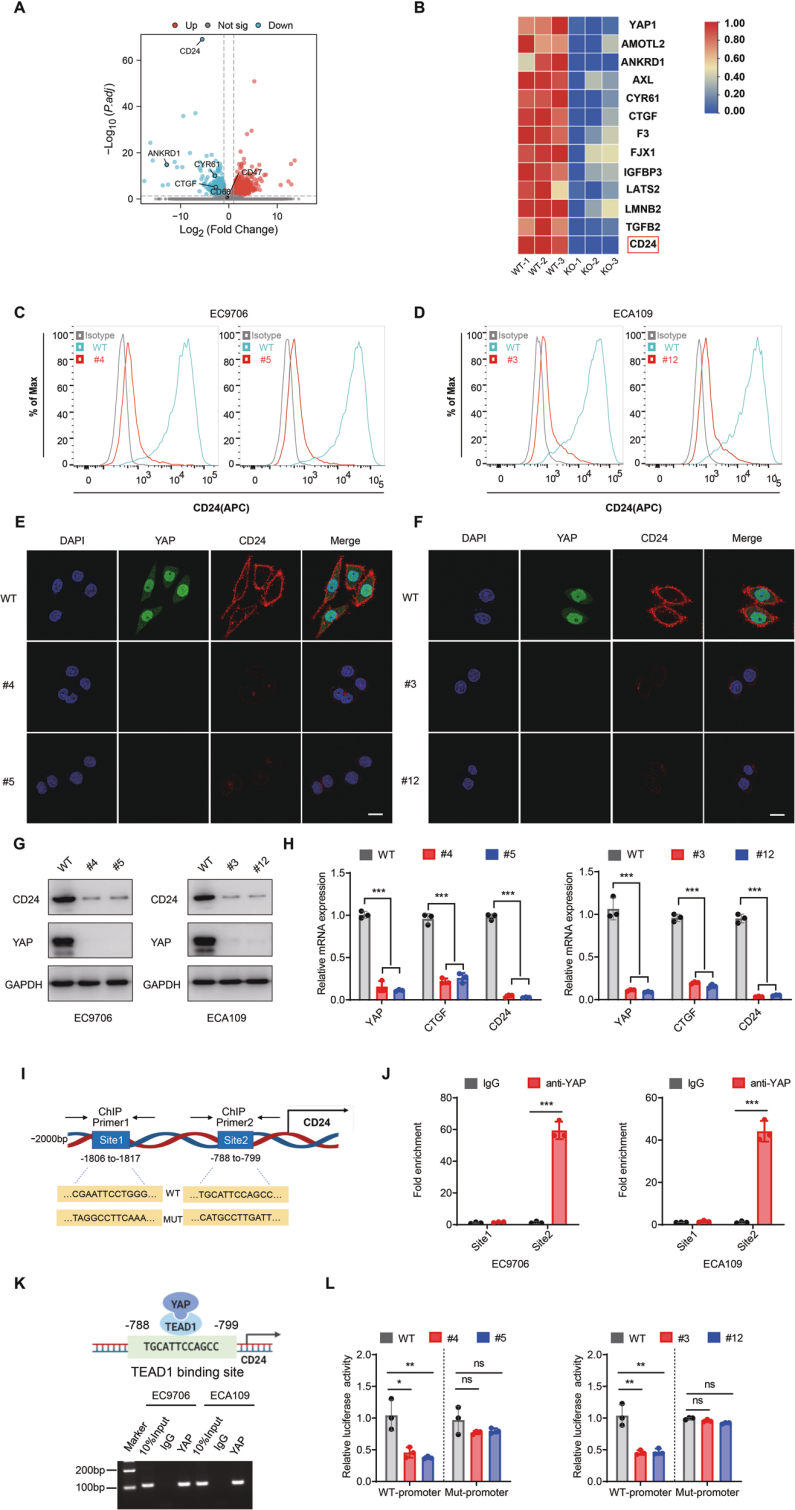
CD24 is direct target gene of YAP. **A** RNA-seq results showed that the expression of CD24 was regulated by YAP. **B** Volcano plot showed that CD24 was the most significant depleted gene after YAP knockout. **C**–**F** Flow cytometry and immunofluorescence analysis of CD24 cell-surface expression indicated CD24 decreased sharply on the cell membrane in YAP^−/−^ cells. Scale bars, 10 μm. **G** Knockout of endogenous YAP reduced levels of endogenous CD24 protein in different human esophageal squamous cells. **H** YAP knockout decreased the CD24 mRNA level in different human esophageal squamous cell. **I** Schematic of the putative TEAD-binding site in the CD24 promoter and the primers used for chromatin immunoprecipitation (ChIP) analysis. Highlighted sequences are wild-type (WT) and two mutated (Mut) CD24 promoter luciferase constructs. **J** ChIP-qPCR analysis of ESCC cells with anti-YAP antibody or IgG control. **K** ChIP-PCR analysis of YAP in the ESCC cells indicates that YAP was enriched at the CD24 promoter. **L** Luciferase reporter assays in WT and YAP KO cells transfected with CD24 promoter reporter containing wild-type (WT) or a mutated TEAD-binding site (MUT).

To further investigate the mechanism underlying the role of YAP in tumor progression, we carried out an RNA-seq analysis in YAP WT and KO cells. The RNA-seq results showed that well-known YAP target genes such as CTGF, CYR61, and AMOTL2 [31] were significantly downregulated after YAP knockout (Fig. 2B). Notably, among the differentially expressed genes identified in our RNA-seq results, we noted that expression of CD24 was the most statistically significant down regulated after YAP depletion (Fig. 2A). CD24 is a novel “don’t eat me” signal and is used by cancer cells to protect themselves from phagocytosis by macrophages. Cell-surface expression of CD24 is required for its antiphagocytic capacity, so flow cytometry and confocal microscopy was performed to determine the degree of CD24 expression. Compared with the corresponding WT subclones, the cell-surface expression of CD24 in EC9706 and ECA109 YAP^−/−^ subclones decreased sharply (Fig. 2C–F). Next, we examined CD24 mRNA and protein expression in YAP^−/−^ cells. As expected, the mRNA and protein expression levels of CD24 in YAP knockout cells were significantly decreased compared to WT cells (Fig. 2G–H). Collectively, these results suggest CD24 may be an important downstream target of YAP in ESCC.

### CD24 is a novel direct target of YAP

As a transcription co-activator, YAP isn’t able to bind DNA directly but mainly interacts with the TEAD family transcription factors to regulate target gene expression [32, 33]. We therefore asked whether CD24 could be transcriptionally regulated by TEAD. First, we used the bioinformatics analyses (JASPAR and LASAGNA algorithm) and predicted that transcription factor TEAD1 had two binding sites for the CD24 promoter, “TGCATTCCAGCC” (Site 1) and “CGAATTCCTGGG” (Site 2) (Fig. 2I). To determine whether YAP is recruited to the CD24 promoter in ESCC cells, chromatin immunoprecipitation (ChIP) assays were performed in EC9706 and ECA109 cells. According to the previously predicted binding sites, we designed two primer pairs, and the ChIP results showed that YAP could directly bind to the CD24 promoter at Site 2 (Fig. 2J, K). To further analyze whether YAP regulates CD24 expression at the transcriptional level, dual-luciferase reporter assays were performed. The luciferase reporter assay showed that YAP depletion reduced the luciferase activity of the CD24 promoter containing the WT binding Site 2, whereas CD24 promoter containing mutant Site 2 did not (Fig. 2L). We further investigated whether the similar results were obtained after TEAD1 silencing in ESCC cells. As expected, TEAD1 silencing led to inhibition of CD24 transcriptional activity (Supplementary Fig. 2A, B and Supplementary Table 1). Since cell-cell contact at high-cell density strongly suppresses YAP activity [34], we reseeded ESCC cells to either sparse or dense conditions. Consistently, the levels of CTGF (a well-established target gene of YAP) and CD24 were decreased in densely cultured cells compared to sparsely cultured cells (Supplementary Fig. 2C and Supplementary Fig. 2G, H). In addition, similar to YAP knockout, treatment with verteporfin, the inhibitor of the interaction of YAP with TEAD [35], also led to downregulation of CD24 in ESCC cells (Supplementary Fig. 2D–F). Collectively, our data suggest that YAP directly regulates CD24 transcription through TEAD-binding to the CD24 promoter.

### YAP-deficiency significantly promotes phagocytosis of ESCC cells by macrophages

**Fig. 3:**
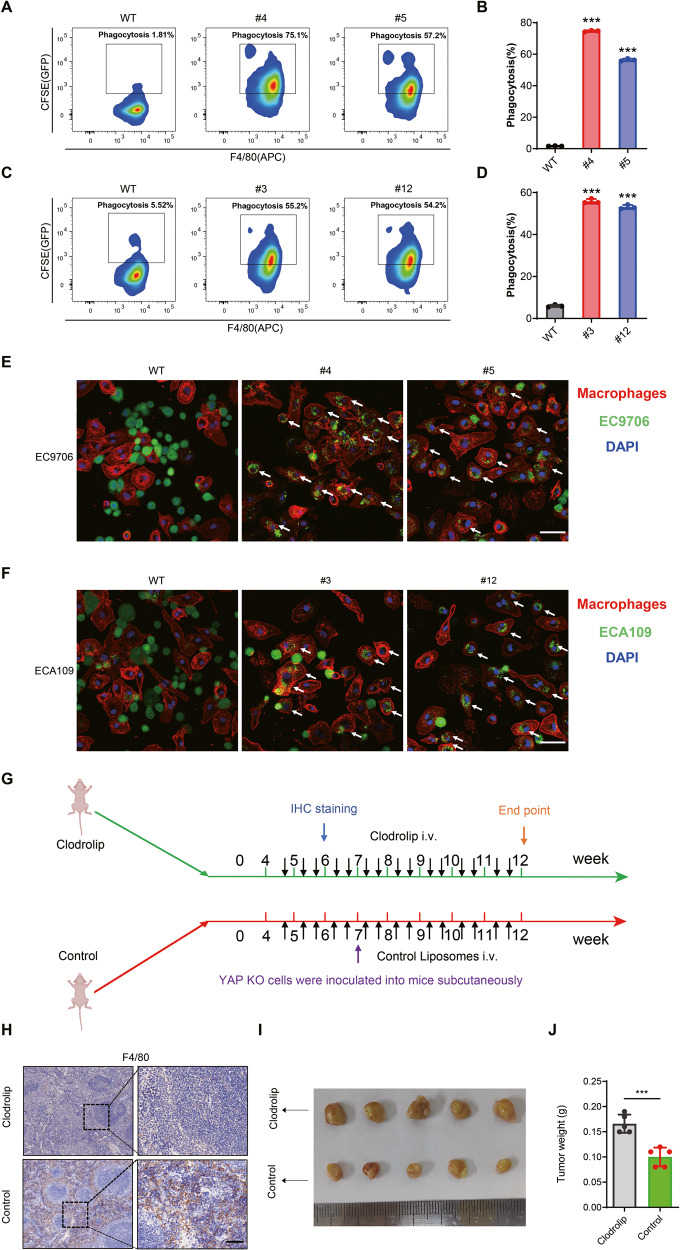
YAP-deficiency promoted phagocytosis of tumor cells by macrophages. **A**, **C** WT and YAP^−/−^ ESCC cells were stained with CFSE, incubated with mouse bone marrow-derived macrophages for 2 h, then stained with F4/80-APC antibody and analyzed by flow cytometry. The result show significantly higher phagocytosis of YAP^−/−^ cells compared with the WT cells. **B**, **D** Statistical analysis of phagocytosis of ESCC cells by BMDMs. Phagocytosis was described as the percentage of F4/80+ GFP+ phagocytosed cancer cells by F4/80+ macrophages. **E**, **F** Representative fluorescence microscopy images of in vitro phagocytosis of WT and YAP^−/−^ cells (CFSE; green) by macrophages (F4/80; red). The white arrows point to macrophages that phagocytose cancer cells. Scale bar, 100 μm. **G** Schematic timeline illustrating the establishment of the xenograft model for macrophage depletion. Colored arrows indicate the time when different events occurred. **H** Macrophage depletion was confirmed by F4/80 immunohistochemical staining in the spleen. Scale bar, 50 μm. **I**, **J** In the presence of macrophages, the tumor formation of YAP knockout cells was significantly restricted.

CD24 is known to be a “don’t eat me” signal and plays an important role in regulating the phagocytosis of cancer cells by macrophages. We demonstrated that the expression of CD24 on the surface of YAP deficient human esophageal squamous cells decreased significantly; we hypothesized that YAP inhibition would promote the phagocytosis of ESCC cells by macrophages. To test this hypothesis, we performed in vitro phagocytosis assays on EC9706 and ECA109 subclones. First, mouse bone marrow cells were isolated and differentiated into bone marrow-derived macrophage (BMDM) cells that were >95% positive for CD11b and F4/80 (Supplementary Fig. 3A). The ESCC cells were labeled with CFSE, cocultured with BMDM for 2 h, then incubated with APC-labeled F4/80 antibody. The cells were analyzed by flow cytometry to detect APC+ CFSE+ cells, which represent macrophages that have phagocytized ESCC cells [36, 37]. The gating strategy is shown in Supplementary Fig. 3B. The results showed that compared with the respective WT subclone, phagocytosis was significantly increased in EC9706 (Fig. 3A, B) and ECA109 (Fig. 3C, D) YAP^−/−^ clones. Meanwhile, substantial whole cell phagocytosis was observed by confocal microscopy in YAP^−/−^ cells, but wild-type cells were rarely engulfed (Fig. 3E, F). The confocal microscopy results were fully consistent with those obtained by flow cytometry. Additionally, we also isolated human peripheral blood-derived macrophages and conducted phagocytosis experiments. The results showed that human peripheral blood-derived macrophages yielded similar experimental outcomes as mouse BMDM (Supplementary Fig. 3C–F). Since YAP also have a cancer cell-intrinsic function, we established a xenograft model of TAM depletion using clodronate. The xenograft results showed that in the presence of macrophages, the tumor formation of YAP knockout cells was significantly restricted compared to the clodronate group (Fig. 3G–J). These results suggest that YAP plays critical roles in regulating the phagocytic activity of macrophages.

### YAP activation induces CD24 expression in ESCC cells

**Fig. 4:**
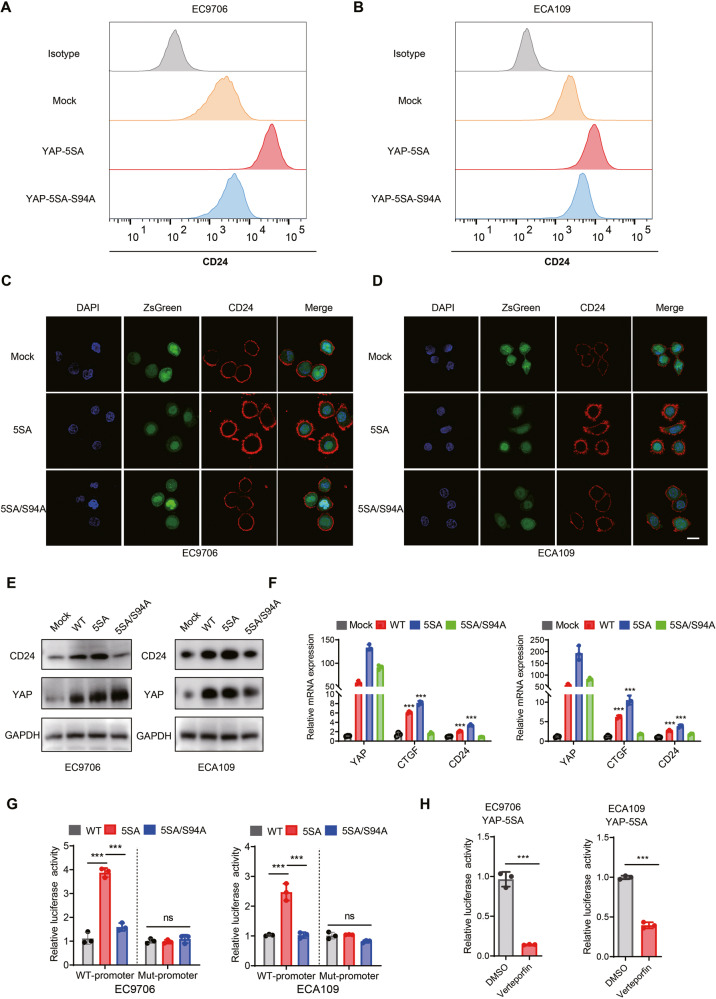
YAP activation induces CD24 expression in ESCC cells. **A**, **B** Flow cytometry analysis of CD24 cell surface expression in Mock, YAP-5SA, and YAP-5SA-S94A expressing EC9706 and ECA109 cells. **C**, **D** Immunofluorescence analysis of CD24 cell surface expression in Mock, YAP-5SA, and YAP-5SA-S94A expressing EC9706 and ECA109 cells. Scale bars, 10 μm. **E** Immunoblotting analysis shows constitutive YAP activation induced CD24 expression in ESCC cells. **F** qRT-PCR analysis of CD24 mRNA levels in Mock, YAP-WT, YAP-5SA, and YAP-5SA-S94A expressing EC9706 and ECA109 cells. **G** Luciferase reporter activity was measured in cells expressing the indicated plasmids. **H** Blocking YAP activation by VP resulted in lower CD24 expression.

The reduction of CD24 expression by depletion of YAP has been confirmed. Next, we examined the role of activated YAP on CD24 expression in ESCC cells. Using lentiviral infection, we established stably transduced ESCC cells that overexpressed YAP-5SA (constitutively active YAP that cannot be phosphorylated by LATS kinases) and YAP-5SA-S94A (the TEAD-binding deficient YAP) [38]. Interestingly, ESCC cells expressing YAP-WT and YAP-5SA displayed greater CD24 expression when compared with mock-infected cells, while YAP-5SA-S94A lost its ability to induce CD24 expression based on flow cytometry (Fig. 4A, B). Similar findings were also verified by immunofluorescence analysis (Fig. 4C, D), WB analysis (Fig. 4E), and qRT-PCR analysis (Fig. 4F). In addition, the luciferase reporter assay showed that YAP 5SA enhanced CD24 promoter activity, whereas YAP-5SA-S94A did not. It is worth noting that both YAP 5SA and YAP-5SA-S94A did not affect the luciferase activities at mutant putative sites on the CD24 promoter (Fig. 4G). Moreover, treatment of YAP-5SA-expressing cells with VP significantly attenuated the transcriptional activation of CD24 (Fig. 4H and Supplementary Fig. 4A, B). These results suggest that YAP activation is crucial for CD24 expression.

### YAP activation inhibits the phagocytosis of ESCC cells by macrophages

**Fig. 5:**
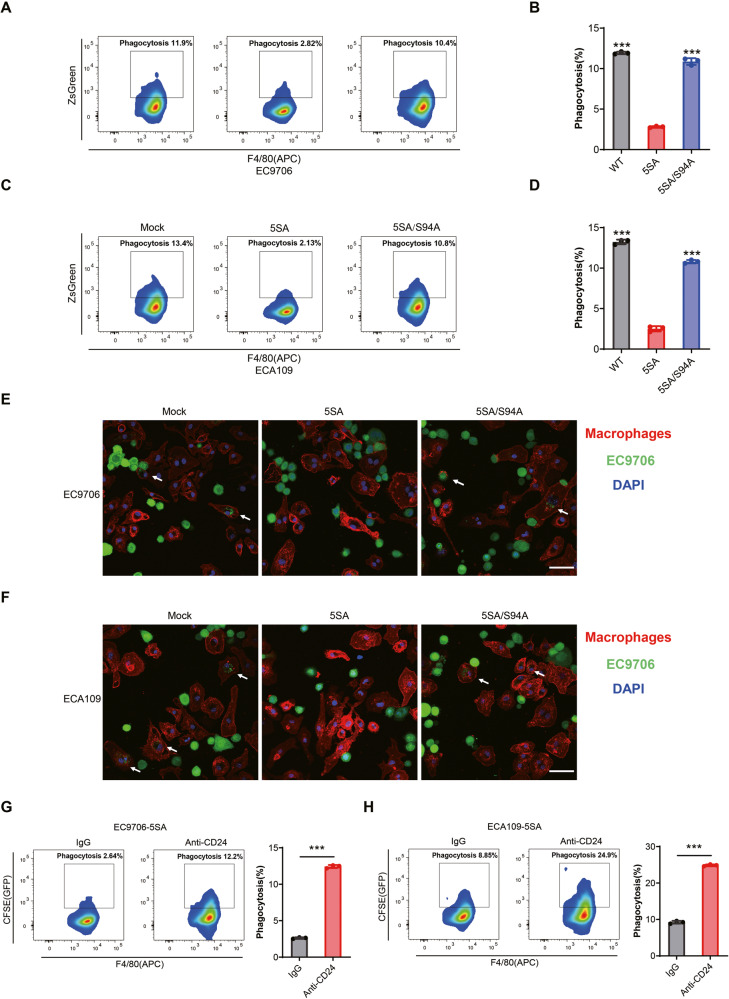
YAP activation inhibited phagocytosis of tumor cells by macrophages. **A**, **C** Mock, YAP-5SA, and YAP-5SA-S94A cells with ZsGreen fluorescence were incubated with mouse bone marrow-derived macrophages for 4 h, then stained with F4/80-APC antibody, and analyzed by flow cytometry. YAP 5SA, but not YAP 5SA/S94A, had an enhanced ability to resist phagocytosis by macrophages. **B**, **D** Statistical analysis of phagocytosis of ESCC cells by BMDMs. Phagocytosis was described as the percentage of F4/80 + GFP+ phagocytosed cancer cells by F4/80+ macrophages. **E**, **F** Representative fluorescence microscopy images of in vitro phagocytosis of Mock, YAP-5SA, and YAP-5SA-S94A cells (ZsGreen; green) by macrophages (F4/80; red). The white arrows point to macrophages that phagocytose cancer cells. Scale bar, 100 μm. **G**, **H** Blockade of CD24 by using anti-CD24 monoclonal antibodies increased macrophage-mediated phagocytosis in YAP-activated ESCC cells.

We found that YAP regulated the expression of CD24 and played an important role in the phagocytosis of tumor cells by macrophages. To further evaluate the possible regulatory role of YAP in phagocytosis, we performed in vitro phagocytosis experiments with YAP-5SA or 5SA/S94A-overexpressing ESCC cells. ESCC cells expressing ZsGreen were cocultured with BMDM for 4 h, then incubated with APC-labeled F4/80 antibody. The cells were analyzed by flow cytometry to detect APC+ ZsGreen+ cells, which represent macrophages that have phagocytosed ESCC cells. As expected, overexpression of YAP-5SA, but not the TEAD-binding deficient YAP (5SA/S94A), inhibited phagocytosis of ESCC cells by macrophages (Fig. 5A–D). Consistent with the results of flow cytometry, resistance to phagocytosis was also shown for YAP-5SA-expressing cells by confocal imaging (Fig. 5E, F). Furthermore, we performed in vitro phagocytosis assays by incubating BMDMs with YAP-5SA overexpressing ESCC cells, with or without CD24 blocking. As expected, CD24 blockade increased the phagocytosis of cancer cells by macrophages in vitro, as shown in the flow cytometry results (Fig. 5G, H). These findings indicate that YAP activation specifically inhibited macrophage-phagocytic activity in a CD24-dependent manner.

### Treatment with Verteporfin suppressed tumorigenesis and progression of ESCC in vivo

**Fig. 6:**
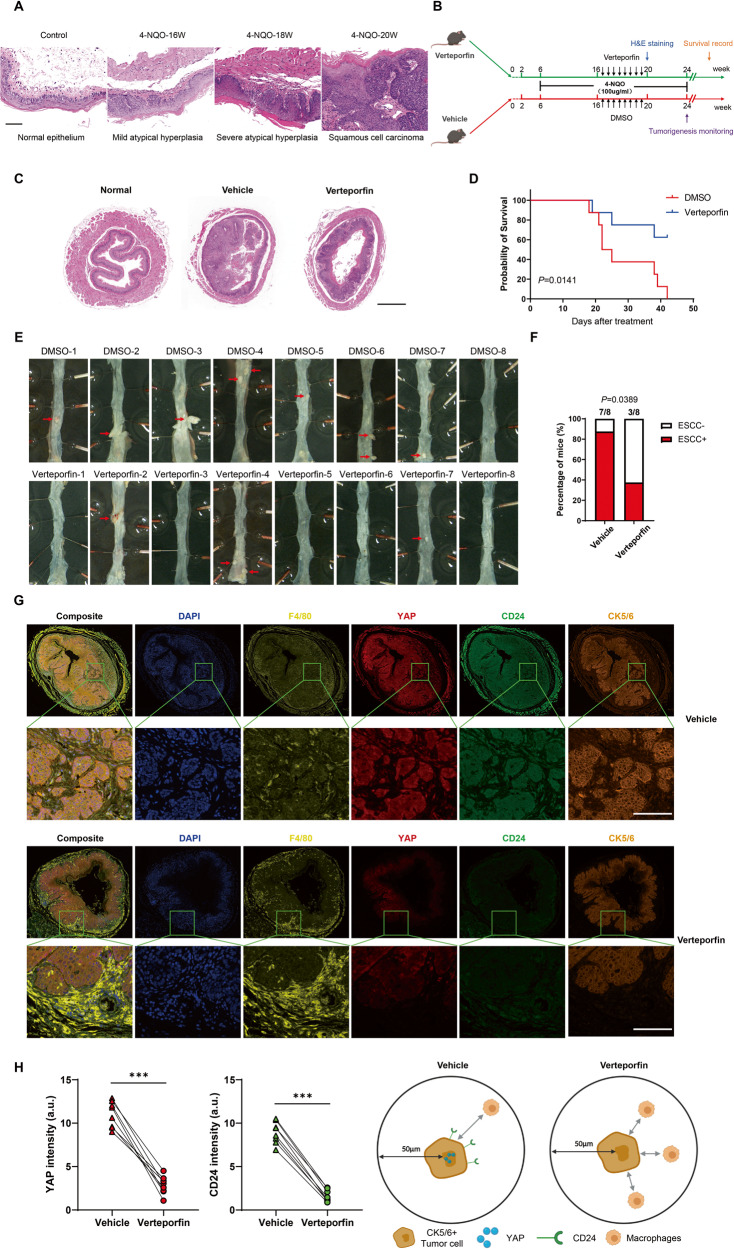
Treatment with YAP selective inhibitor suppressed tumorigenesis and progression of ESCC in vivo. **A** Pathological features of normal esophagus and esophagus tissues at different time points after 4-NQO induction. Scale bars, 100 μm. **B** Schematic diagram of the timeline of establishing the 4-NQO-induced mouse model of ESCC. Colored arrows indicate the time when different events occurred. **C** Morphological images of esophagus collected from both the control and Verteporfin-treated mice after administration of eight doses of VP. Verteporfin treatment reduced tumor incidence in the ESCC mouse model. Scale bar, 500 μm. **D** Kaplan–Meier analysis showing significantly longer survival times in Verteporfin-treated mice (n = 8) than in control mice (*n* = 8). **E** Morphological images of esophagus collected from VP treated and control mice after VP withdrawal for 4 weeks. **F** The incidence of ESCC in the VP group was lower than in the control group. **G** mIHC staining of F4/80 (yellow), YAP (red), CD24 (green) and CK5/6 (orange) in mouse ESCC demonstrated the YAP selective inhibitor could affect the spatial distribution of macrophages (*n* = 8). Representative images are shown. **H** Quantitative and spatial analysis showed that the expression of YAP and CD24 decreased significantly after VP treatment, and significantly more macrophages could be found in close proximity (≤50 µM radial distance) of CK5/6+ cells which have a low YAP-CD24 expression.

The 4-nitroquinoline-1-oxide (4-NQO) induced ESCC model in mice has been proven to be a reliable and reproducible system by our group and other researchers [39–41]. According to the histological analysis of induced mouse esophageal epithelium, we found that mild atypical hyperplasia began to appear in the esophagus of induced mice at 16 weeks, and developed into esophageal squamous cell carcinoma at 20 weeks (Fig. 6A). Atypical hyperplasia of the esophageal squamous epithelial tissues is considered to be a precancerous condition in which the accumulated cells transform into cancerous cells. We started treatment with verteporfin (VP;100 mg/kg i.p. twice a week) at 16 weeks, and pathological analysis was performed after eight doses of VP in mice (*n* = 8). Another group of mice were sacrificed at 24 weeks (*n* = 8), and esophageal tissue was macroscopically examined and the number of macroscopic lesions was counted. The rest of the experimental design is summarized in Fig. 6B. The results showed that VP treatment markedly suppressed the progression of ESCC in mice (Fig. 6C and Supplementary Fig. 6), and VP treatment significantly reduced tumor multiplicity compared to vehicle treatment (Fig. 6E, F). In addition, mice that received VP treatment had better survival than the vehicle control group (Fig. 6D). Next, we assessed if tumor YAP expression was associated with the macrophage infiltration in the mIHC. As expected, mIHC data indicated that the intensity of YAP and CD24 expression decreased significantly after VP treatment (Fig. 6G). Besides, spatial analysis showed that significantly more macrophages could be found in close proximity (≤50 µM radial distance) of CK5/6+ cells which have a low YAP-CD24 expression (Fig. 6H), implying a potential regulatory role of YAP-CD24 axis in macrophage-mediated tumor immunity.

Additionally, we observed similar results in the histological analysis of xenograft tumors: the expression of YAP and CD24 was significantly reduced in the KO group compared to the WT group. Notably, while performing F4/80 staining, we observed a substantial recruitment of macrophages in the KO group, primarily concentrated around the tumor. However, the WT group with a heavier tumor burden displayed limited presence of macrophages. In addition, we did not observe a significant difference in the M1 marker iNOS. However, compared to the KO group, the WT group exhibited significantly higher expression of the M2 marker, CD163(Supplementary Fig. 5).

### Clinical significance of YAP and CD24 expression in ESCC

**Fig. 7:**
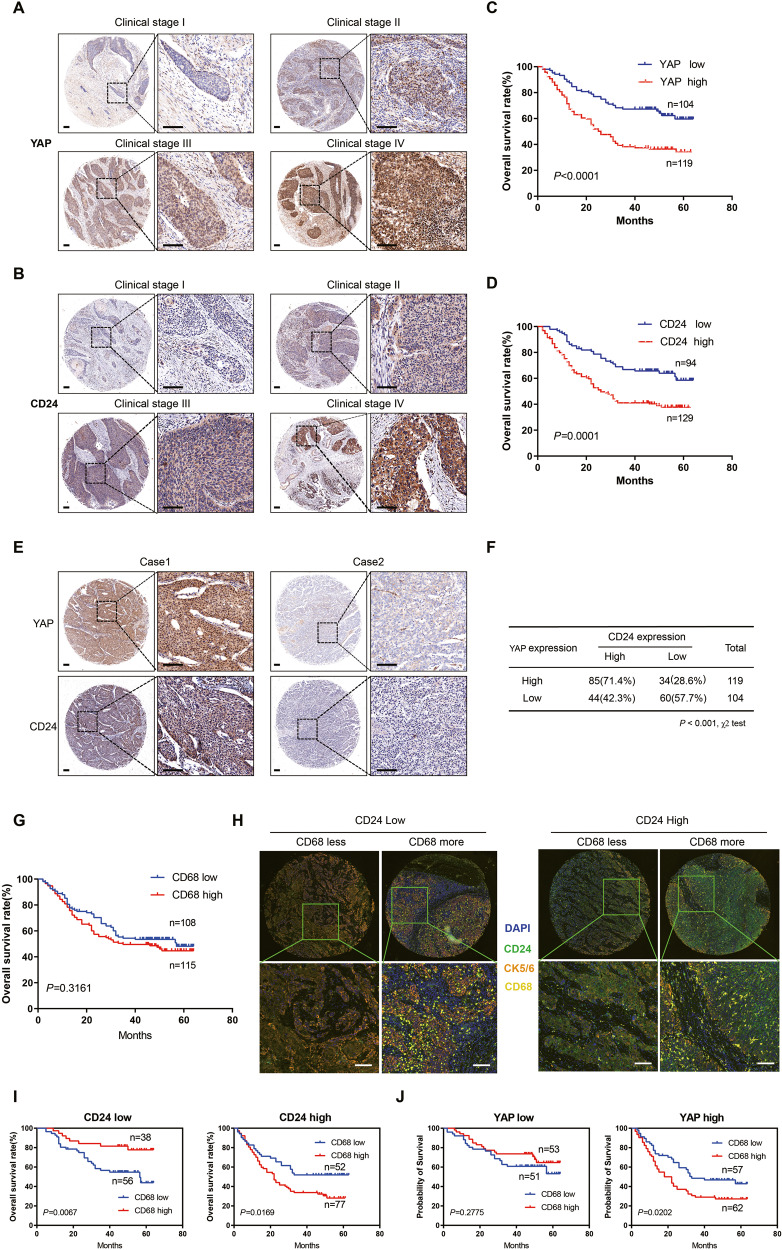
Clinical significance of YAP-CD24 axis in ESCC. **A**, **B** Overexpression of YAP and CD24 was significantly correlated with advanced clinical stages in ESCC. Scale bars, 100 μm. **C**, **D** Kaplan–Meier analysis revealed that higher expression of YAP and CD24 was related with poorer overall survival of ESCC patients. *P* < 0.001, log-rank test. **E**, **F** Expression of YAP was positively correlated with CD24 in human ESCC. **G** Kaplan−Meier analysis shows that the expression of CD68 was not associated with the overall survival rate of ESCC patients. **H** Representative multiplexed IHC images of ESCC tissue. The panel of biomarkers and their corresponding pseudo-colors are the following: CD24 (green), CD68 (yellow), CK5/6 (orange), and DAPI (blue). Scale bars, 100 μm. **I** Kaplan–Meier analyses of OS according to CD68+ macrophage infiltration in low (left, *n* = 94) and high (right, *n* = 129) CD24 expression subgroups. Data were analyzed by log-rank test. **J** Kaplan–Meier analyses of OS according to CD68+ macrophage infiltration in low (left, *n* = 104) and high (right, *n* = 119) YAP expression subgroups. Data were analyzed by log-rank test.

Next, we explored the clinical significance of YAP and CD24 expression in ESCC. The levels of YAP and CD24 proteins were determined by IHC staining using a human tissue microarray (TMA) containing 223 cases of ESCC tissues. As shown in Supplementary Table 2, high expression of YAP was significantly associated with lymph node metastasis (*P* < 0.001), advanced clinical stage (*P* < 0.001; Fig. 7A), and tumor invasion (*P* = 0.0319), while high expression of CD24 was significantly associated with lymph node metastasis (*P* = 0.0024), advanced clinical stage (*P* < 0.001; Fig. 7B), distant metastasis (*P* = 0.01), and tumor invasion (*P* = 0.0095). Kaplan–Meier analysis indicated that high YAP expression and high CD24 expression were correlated with worse overall survival (log-rank test: both *P* < 0.001; Fig. 7C, D). In the univariate analysis, differentiation (*P* = 0.015), tumor invasion (*P* = 0.001), lymph node metastasis (P < 0.001), distant metastasis (*P* < 0.001), clinical stage (*P* < 0.001), YAP expression (*P* < 0.001), and CD24 expression (*P* < 0.001) were the statistically significant predictors for a patient’s overall survival (Table 1). Multivariate Cox proportional regression analysis further revealed that differentiation (*P* = 0.013), clinical stage (*P* = 0.013), YAP expression (*P* = 0.026), and CD24 expression (*P* = 0.009) were independent prognostic factors for the overall survival of ESCC patients (Table 1).

**Table 1:** Cox proportional hazard regression analyses for overall survival.

Clinical characteristics	Univariable analysis		Multivariable analysis	
	HR^a^ (95% CI^b^)	*P* value	HR (95% CI)	*P* value
Age	0.848 (0.581–1.239)	0.395		
Gender	1.028 (0.696–1.517)	0.890		
Differentiation	1.579 (1.091–2.284)	**0.015**	1.613 (1.108–2.351)	**0.013**
Tumor invasion	2.033 (1.326–3.116)	**0.001**	1.119 (0.685–1.828)	0.654
Lymph node metastasis	3.556 (2.450–5.163)	<**0.001**	1.253 (0.656–2.396)	0.494
Distant metastasis	4.084 (2.487–6.708)	**<0.001**	1.710 (0.983–2.978)	0.058
Clinical stage	4.047 (2.778–5.895)	<**0.001**	2.509 (1.212–5.195)	**0.013**
YAP expression	2.272 (1.538–3.358)	**<0.001**	1.601 (1.059–2.421)	**0.026**
CD24 expression	2.145 (1.439–3.196)	**<0.001**	1.723 (1.114–2.595)	**0.009**

^a^Hazard ratio.

^b^Confidence interval.

Bold values indicates statistical significance (*P* < 0.05).

### The relationship between YAP, CD24, and CD68+TAMs in ESCC

The correlation between YAP expression and CD24 levels was further investigated in human ESCC specimens in TMA. As shown in Fig. 7E, F, we found that elevated YAP expression highly correlated with CD24 expression in ESCC tissues (*P* < 0.001). Up to now, the relationship between the infiltration of CD68+TAMs in ESCC and prognosis has been controversial and contradictory [42–45]. In our study, we found that CD68+TAMs infiltration was not associated with OS in ESCC patients (Fig. 7G). However, we observed that ESCC patients with low expression of CD24 and high CD68+ macrophage infiltration had better overall survival (OS), while ESCC patients with high expression of CD24 and high CD68+ macrophage infiltration had worse overall survival (Fig. 7H–I). These data suggest that if we divide ESCC patients into high and low CD24 expression groups, the impact of macrophages on prognosis will undergo dramatic changes. Similar findings were also observed when ESCC patients were divided into high and low YAP expression groups, although the effect was not statistically significant in the low-YAP expression group (Fig. 7J). These results suggest that the “don’t eat me” signal CD24 plays a key role in TAMs affecting the prognosis of patients.

## Discussion

Evasion from immune destruction is a critical hallmark of cancer [46]. By utilizing immune checkpoint signaling pathways, cancer cells can send signals to anti-tumor immune cells and escape immune surveillance. Growing evidence indicates that targeting TAMs to induce TAMs to phagocytose cancer cells is a promising therapeutic strategy [47]. CD47 is a well-known “don’t eat me” signal, which binds to signal regulatory protein α (SIRPα) to regulate macrophage phagocytosis [48]. Based on findings from preclinical studies and early clinical data, blocking the “don’t eat me” signal CD47 has the potential to benefit patients with advanced malignancies [49]. However, due to the high expression of CD47 in human red blood cells and platelets, blocking CD47 causes side effects such as severe anemia and thrombocytopenia, which greatly limits the clinical development of such therapeutics. CD24 is a novel “don’t eat me” signal which can play an immune escape function by interacting with TAM receptor Siglec-10. In some cases, CD24 may act as a complementary signal for CD47 and seems to have inversely correlated expression in human tumors. Moreover, unlike blocking CD47, which is more sensitive in hematological malignancies, blocking CD24 is more sensitive in solid tumors such as ovarian and triple‐negative breast cancers [50]. Overall, CD24 is a promising new target of immunotherapy, and its regulatory mechanism needs to be further studied.

CD24 has long been considered a regulator of cell migration, invasion, and adhesion [51, 52]. CD24 on tumor cells is known to interact with Siglec-10 on innate immune cells to inhibit engulfment. We examined cell-surface expression of CD24 in different ESCC cell lines by flow cytometry. Elevated CD24 expression was observed in 6/7 ESCC cell lines when compared with an immortalized esophageal epithelial cell line (HEEC) (Supplementary Fig. 7A). In addition, through mining TCGA esophageal cancer data, we found that CD24 expression was significantly upregulated in esophageal carcinoma tissues (Supplementary Fig. 7B). Therefore, CD24 may serve as an attractive target for cancer therapy.

In contrast to another report, our overall experimental results indicate that the Hippo-YAP signaling axis has a positive regulatory effect on CD24 [53]. We found that YAP directly regulates CD24 transcription through TEAD-binding to the promoter region of CD24. This difference may be attributed to variances in the tumor types and/or cell lines selected in these studies. Our results show that YAP inactivation downregulates CD24 expression in ESCC cells. Conversely, the activation of YAP leads to a strong induction of CD24 expression. We then investigated whether YAP could impact the phagocytosis of macrophages through the YAP-CD24 axis. Intriguingly, we found that the phagocytic activity of macrophages toward tumor cells increased after YAP deletion, while the phagocytic activity of macrophages toward tumor cells was inhibited after YAP activation. Furthermore, the effects of inhibiting macrophage-phagocytic activity after YAP activation can be reversed by blocking CD24. As described previously, CD47 is an important “don’t eat me” signal, we further examined the effect of YAP knockout/activation on the expression level of CD47, the results showed the expression of CD47 was not associated with YAP depletion or activation (Supplementary Fig. 8A-D). These results suggest that YAP could serve as an important regulator of tumor immunity through the “don’t eat me” signal CD24 in ESCC.

Hippo signaling plays a critical role in promoting the tumorigenesis and metastasis of human tumors. YAP is the core effector of the Hippo pathway, which interacts with the corresponding transcription factors to activate transcription of downstream genes. In different conditions, YAP can function as both an oncogene and a tumor suppressor gene. There is a report suggesting that YAP functions as a tumor suppressor, whereas TAZ plays a role in promoting tumor growth in ESCC [54]. However, our data and other studies from different teams do not support this view. On one hand, through the GSEA analysis of RNA-seq data, we found significant enrichment of E2F target genes, G2M checkpoint genes and Epithelial–Mesenchymal Transition genes in WT cells rather than KO cells after YAP knockout. The WB results showed a significant decrease in these candidate genes after YAP knockout, consistent with the RNA-seq results (Supplementary Fig. 9). On the other hand, we further knocked down the TAZ gene in ESCC cell lines and observed a decrease in the proliferation and migration abilities of the tumor cells. This suggests that TAZ and YAP have similar roles and play a certain driving role in ESCC (Supplementary Fig. 10). In fact, overexpression or activation of YAP is very common in ESCC, and YAP expression is significantly associated with clinical stage and poor prognosis in ESCC [55]. Previous studies of YAP mostly focused on the regulation of tumor cells themselves, such as proliferation, migration, invasion, and stemness. Indeed, YAP has been shown to be an oncoprotein in ESCC in quite a few studies and the mechanism of YAP activation controlled by the Hippo pathway has been gradually clarified [56]. However, the limited success of targeting only tumor cells themselves in clinical trials highlights the importance of extending treatment strategies to the TME [57]. Interestingly, more and more studies have demonstrated that YAP is also involved in the regulation of TME. For example, YAP promoted the differentiation of Treg and exerted an immunosuppressive function in tumor microenvironment [58]. In addition, YAP has been reported to affect the recruitment and polarization of TAMs, although the precise mechanism has still not been completely elucidated [59].

In this study, we found for the first time that the Hippo signaling pathway regulates the phagocytic activity of macrophages by regulating CD24. Our findings delineate a previously unrecognized signaling axis from Hippo, YAP, and TEAD to the transcriptional activation of CD24 through regulation of the CD24 promoter. This model advances our mechanistic understanding of the role of YAP, which has previously been shown to regulate development, organ size, regeneration, and tumorigenesis. Additionally, our work links the Hippo pathway with macrophage-mediated immune escape, which is a major addition to the Hippo pathway. Furthermore, the YAP inhibitor selected in this study is Verteporfin, an early-identified small molecule compound that can act as an inhibitor of YAP-TEAD interaction and is commonly used as a tool compound for Hippo pathway research [58, 60–62]. Although our preliminary experimental results show that Verteporfin significantly reduces the mRNA and protein levels of YAP, its off-target effects still need to be considered. Therefore, further research is needed to confirm the effectiveness of YAP/TEAD inhibitors with better tolerance and higher specificity, such as VT107 and VT3989 [63], which is important for future clinical translation.

Another interesting finding of this study is that the “don’t eat me” signal CD24 plays a pivotal role in the effect of macrophages on the prognosis of ESCC. There is abundant evidence demonstrating that presence of tumor infiltrating T lymphocytes is related to the good prognosis of patients with ESCC [64]. However, the function and prognostic value of macrophages in tumors remains conflicting. As for ESCC, some studies have reported that CD68, a pan-marker of macrophages, was associated with a better prognosis, while others reported the opposite. It has also been suggested that the polarization of macrophages is associated with the prognosis. On the one hand, the role of M1 macrophages in tumors is still controversial, and not all tumor-promoting TAMs have M2-like phenotypes [65]. Therefore, it is urgent to redefine the status of TAMs beyond the M1/M2 dichotomy.

In our study, CD68 was not significantly associated with the prognosis of patients with ESCC (Supplementary Table 3). However, if we group patients according to CD24 expression, the impact of macrophages on the prognosis of patients will changes dramatically. That is, macrophages are associated with a better prognosis when the TME lacks the “don’t eat me” signal, while macrophages are associated with a worse prognosis when the TME highly expresses the “don’t eat me” signal. This partly explains why the impact of macrophages on the prognosis of patients is contradictory, because the “don’t eat me” signal is not generally considered as an evaluation factor. These findings highlight the importance of the “don’t eat me” signal in macrophage function evaluation and provide a strong basis for the development of precision medicine.

Overall, our findings provide new insights for understanding the molecular mechanisms of ESCC, and delineate a potential therapeutic strategy for augmenting macrophage phagocytic activity and improving the prognosis of ESCC.

## Materials and methods

### Cell lines and culture

The normal esophageal squamous cell line HEEC and human esophageal carcinoma cell lines EC9706, ECA109, KYSE140, KYSE150, KYSE450, KYSE70, and KYSE510 were cultured in RPMI 1640 Medium (Sperikon Life Science & Biotechnology co.,ltd) containing 10% fetal bovine serum (CellMax), with 1% penicillin/streptomycin (Sperikon Life Science & Biotechnology co., ltd). HEEC, KYSE140, KYSE150, KYSE450, KYSE70, EC9706, and ECA109 were authenticated by short tandem repeat profiling (STR) (BGI, Shenzhen, China) and they were consistent with cells in China Infrastructure of Cell Line Resources. Cells were regularly tested for mycoplasma contamination using PCR and confirmed to be mycoplasma-free.

### CRISPR/Cas9 knockout and monoclonal cell screening

CRISPR/Cas9 knockout technique was employed to establish YAP null cells. Guide RNA (sgRNA) sequences were designed by Guide Design Resources (http://crispor.tefor.net). Oligo-sequences for sgRNAs are listed in Supplementary Table 4. The annealed double-stranded sgRNA oligonucleotide was cloned into pX458-DsRed2 or pX458-ECFP vectors described previously31. ESCC cells were transfected with each construct containing CRISPR/Cas9 components as well as DsReds or the ECFP reporter using Neon® Transfection System (Thermo Fisher Scientific). Mutant cell populations were selected by FACS sorting technology with a dual fluorescent reporter system. Then, monoclonal cell screening was performed by 96 well plates and verified by PCR amplification and confirmed by Sanger sequencing and Western blot.

### RNA isolation and quantitative real-time PCR (qRT-PCR)

Total RNA was extracted with RNeasy Plus Mini Kit (Qiagen, Valencia, CA). First-strand cDNA was synthesized following the manufacturer’s instructions (Yeasen Biotech Co. Ltd.). Subsequently, 2 μL of diluted cDNA (1:10) was used as a template and the SYBR Green Master Mix (GenStar, China) was used in a final volume of 20 μL for each reaction. A house-keeping gene, GAPDH, was used as an internal control. The sequences of the primers for qRT-PCR are listed in Supplementary Table 5.

### Phagocytosis assay

Bone marrow-derived macrophages (BMDM) were isolated from the femur and tibia of C57BL/6 mice and cultured for 7 days in RPMI-1640 supplemented with L929 cell culture medium (as a source of M-CSF). BMDM were plated (5 × 104 per well) in a 24-well tissue-culture plate. ESCC cells were stained with 2.5 μM carboxyfluorescein succinimidyl ester (CFSE) according to the manufacturer’s protocol (MCE). BMDM were incubated in serum-free medium for 2 h before adding 2 × 105 CFSE-labeled ESCC cells. After coculturing for 2 h at 37 °C, cells were harvested, BMDM were stained with APC-conjugated CD11b (Novus Biologicals), and flow cytometry (FACS canto, BD Biosciences) was performed. Unstained control and single stained cells were prepared for gating.

### Chromatin immunoprecipitation (ChIP) assay

For the ChIP assay, EC9706 and ECA109 cells were crosslinked in 1% formaldehyde for 10 min at 37 °C. Fixed cells were sonicated and the chromatin fragments were subjected to immunoprecipitation using YAP antibody (CST, #14074) or normal rabbit IgG (Santa Cruz Biotechnology, Santa Cruz, CA, USA) according to the instruction of ChIP assay kit (Novus Biologicals, NBP1-71709). The primers encompassing the YAP binding sites in different regions of the CD24 promoter were designed as shown in Supplementary Table 6.

### RNA-seq and analysis

Total RNA was isolated using the RNeasy kit (Qiagen) from three different YAP knockout (KO) clones and three wild-type (WT) EC9706 cells. The RNA samples were subjected to high-throughput RNA sequencing by LC Bio (Zhejiang, China) and data analysis. RNA-seq data are available in the Gene Expression Omnibus repository (GSE201646). Differential gene expression analysis and KEGG enrichment analysis were performed as described by Lc-bio (https://www.lc-bio.cn/).

### Western blot

Cells were lysed in ice-cold Tris buffer (20 mM Tris; pH 7.5) containing a protease inhibitor cocktail (New Cell & Molecular Biotech Co. Ltd) and 1 mM DTT, 10% glycerol, 50 mM NaF, 2 mM ethylenediaminetetraacetic acid, 137 mM NaCl, and 1% Triton X-100 (Solarbio, Beijing, China). The extracted proteins were separated by SDS-PAGE and then electroblotted onto polyvinylidene fluoride membranes (Elabscience Biotechnology Co., Ltd.). The membranes were incubated with antibodies after blocking with NcmBlot Blocking Buffer (New Cell & Molecular Biotech Co. Ltd). Primary antibodies were as follows: YAP (CST, 14074, 1:1000), CD24 (Abways, CY10303,1:500; HUABIO, 0804-3, 1:500) and GAPDH (Solarbio, K200057M, 1:3000). The secondary antibodies were purchased from Lianke Bio and used at a 1:3000 dilution. Protein signals were detected with an ECL kit (Life-iLab, China).

### Luciferase reporter assays

ESCC cells were seeded at a density of 1 × 105 cells/well in 24-well plates 24 h before transfection. Cells were co-transfected with 450 ng/well reporter gene plasmids (pGL3-CD24 and pGL3-CD24-mut) and 50 ng/well pRL-TK control plasmids (Promega) as an internal control reporter. The cells were collected after 36 h and the Luciferase reporter assays were performed using the Dualucif® Firefly & Renilla Assay Kit (UElandy). Each experiment was repeated at least three times.

### Tissue microarray (TMA) and immunohistochemistry (IHC)

A total of 223 cases of ESCC were selected for the TMA construction. All of these tissue samples were obtained from the First Affiliated Hospital of Xinxiang Medical University. No patients recruited in the study received preoperative treatments. All ESCC samples used in this study were authorized by the Committees for Ethical Review of Research at Xinxiang medical University. IHC was performed by Servicebio Biotechnology Co. Ltd. (Wuhan, China) according to standard protocols. YAP antibody (CST, 14074, 1:500), CD163 antibody (Sino Biological, 310222-MM01,1:1000), S100a9 antibody (HUABIO, ET1702-73, 1:100), iNOS antibody (Signalway antibody, 33424-2,1:100), Ly-6G antibody (Biorbyt, orb322983,1:250) and CD24 antibody (HUABIO, 0804-3, 1:200) were used in this study. IHC scores were calculated using the following formula: IHC score = intensity score × percentage score. The intensity score was measured according to the intensity of staining (0: negative, 1: weak, 2: moderate, and 3: strong); the percentage score was determined using the percentage of stained area (0: 0%, 1: 1–25%, 2: 26–50%, 3: 51–75%, and 4: 76–100%). The IHC analysis was performed according to the method we described previously and the characteristics of the patients are summarized in the Supplementary Table 7 [25].

### Animal experiments

Six-week-old female C57BL/6 mice supplied by Vital River Laboratories (city, country), were given 4-NQO (Sigma, N8141) in drinking water (100 μg/mL) to induce ESCC. The mice were randomized into six groups (8/group). To detect the pathological effect of VP (KKL MED, KM12872) treatment on induced mice, VP (100 mg/kg) or vehicle (control) was injected intraperitoneally for 4 weeks and then the mice were euthanized. The tumor incidence and survival time of induced mice were detected in the other four groups at the specified time. All protocols and procedures for animal experiments were reviewed and approved by Ethics Committee of Xinxiang Medical University.

### Multiplexed IHC (mIHC)

mIHC was performed using a multiple fluorescent immunohistochemical staining kit (abs50030, Absin, Shanghai, China) according to the manufacturer’s instructions. In brief, after dewaxing and hydration, microwave antigen repair was carried out. The sections were blocked in 10% normal goat serum and then incubated with the CD24 primary antibodies (Abclonal, A2207, 1:100, TSA520). After washing with TBST, the secondary antibody was incubated, and then the TSA dye was applied for 10 min. This cycle was repeated three more times using the following antibodies: CD68 (Servicebio, GB113150, 1:500, TSA570) or F4/80(Biorbyt, orb555999, 1:1000, TSA570), YAP (CST, 14074, 1:200, TSA700) and CK5/6 (Bioss, bs-20824R,1:100, TSA620). Finally, nuclei were stained with DAPI. Fluorescence images were acquired using the Vectra 3 quantitative pathology imaging system (Akoya Biosciences) and quantified by InForm software (InForm™, PerkinElmer). For spatial interaction analysis, we used the Spatial Analysis Module in HALO v2.0 digital image analysis software (Indica Labs, Corrales, NM), which is compatible with Vectra and InForm software.

### Statistical analysis

No specific statistical tests were used to predetermine the sample size. Statistical analysis was performed using GraphPad Prism 9 (GraphPad Software) or SPSS version 23.0 (SPSS, Inc., IL). All data are presented as means ± s.e.m. Student’s *t*-test was applied to determine the statistical significance of the differences between the two independent groups. Variance is similar between comparison groups. Kaplan-Meier analysis and log-rank test were used to estimate the effect of 2 experimental groups in overall survival (OS). Chi-square tests were used to analyze the association of YAP/CD24 expression with clinic-pathologic factors. Differences between two independent groups were tested with Student’s t-test. Kaplan-Meier analysis with log-rank test was applied for survival analysis. The relation between YAP/CD24 expression and clinicopathological characteristics was analyzed by Pearson χ2 test. Univariate and multivariate Cox proportional hazard regression models were used to evaluate the survival hazard using the Cox proportional hazard model with a forward stepwise procedure.

### Supplementary information

Supplementary Materials for The Hippo-YAP signaling pathway drives CD24-mediated immune evasion in esophageal squamous cell carcinoma via macrophage phagocytosis

Dataset 1

## Data Availability

The authors declare that all data supporting the findings of this study are available within the article and its Supplementary Information files or from the corresponding author upon reasonable request. The experimental data that support the findings of this study are available through FigShare (10.6084/m9.figshare.24770940; 10.6084/m9.figshare.24772566; 10.6084/m9.figshare.24781881).
