# Supplementary material for: The Hippo-YAP signaling pathway drives CD24-mediated immune evasion in esophageal squamous cell carcinoma via macrophage phagocytosis
Source: Oncogene. 2024 Jan 2;43(7):495–510. doi: 10.1038/s41388-023-02923-z (PMC10857940; doi:10.1038/s41388-023-02923-z)
Supplement: Supplementary file 1 — Supplementary Materials for The Hippo-YAP signaling pathway drives CD24-mediated immune evasion in esophageal squamous cell carcinoma via macrophage phagocytosis [file 41388_2023_2923_MOESM1_ESM.docx]

Supplementary Materials for

The Hippo-YAP signaling pathway drives CD24-mediated immune evasion in esophageal squamous cell carcinoma via macrophage phagocytosis


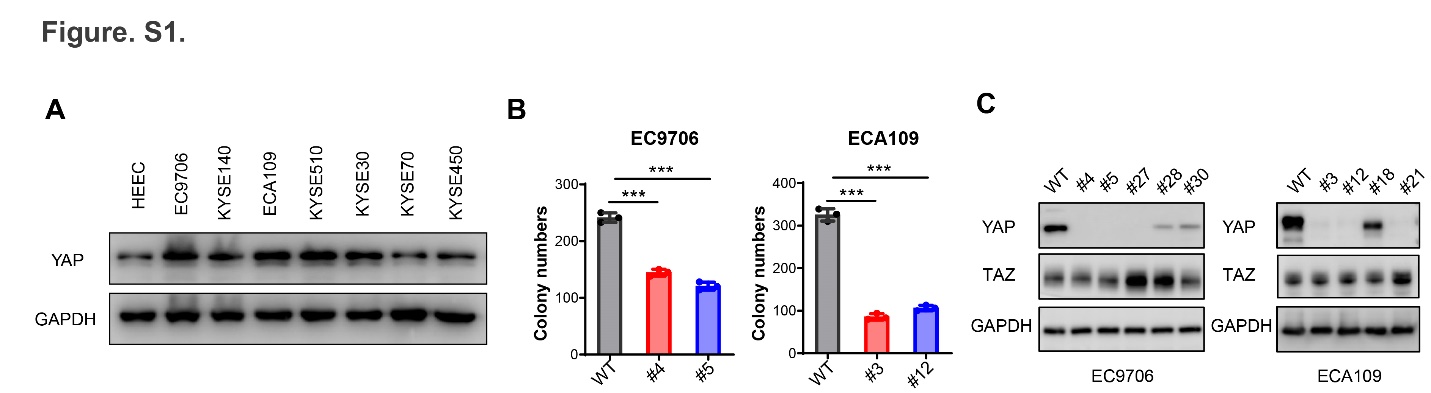


**Figure. S1.**

**Expression of YAP in different esophageal cancer cell lines compared with an immortalized esophageal epithelial cell line (HEEC)**

1. Western blot analysis of YAP expression in ESCC cell lines and one immortalized esophagus cell line (HEEC).
2. Statistical analysis of the results from the colony formation assay.
3. Western blot analysis of YAP and TAZ expression in different subpopulations of monoclonal cells.


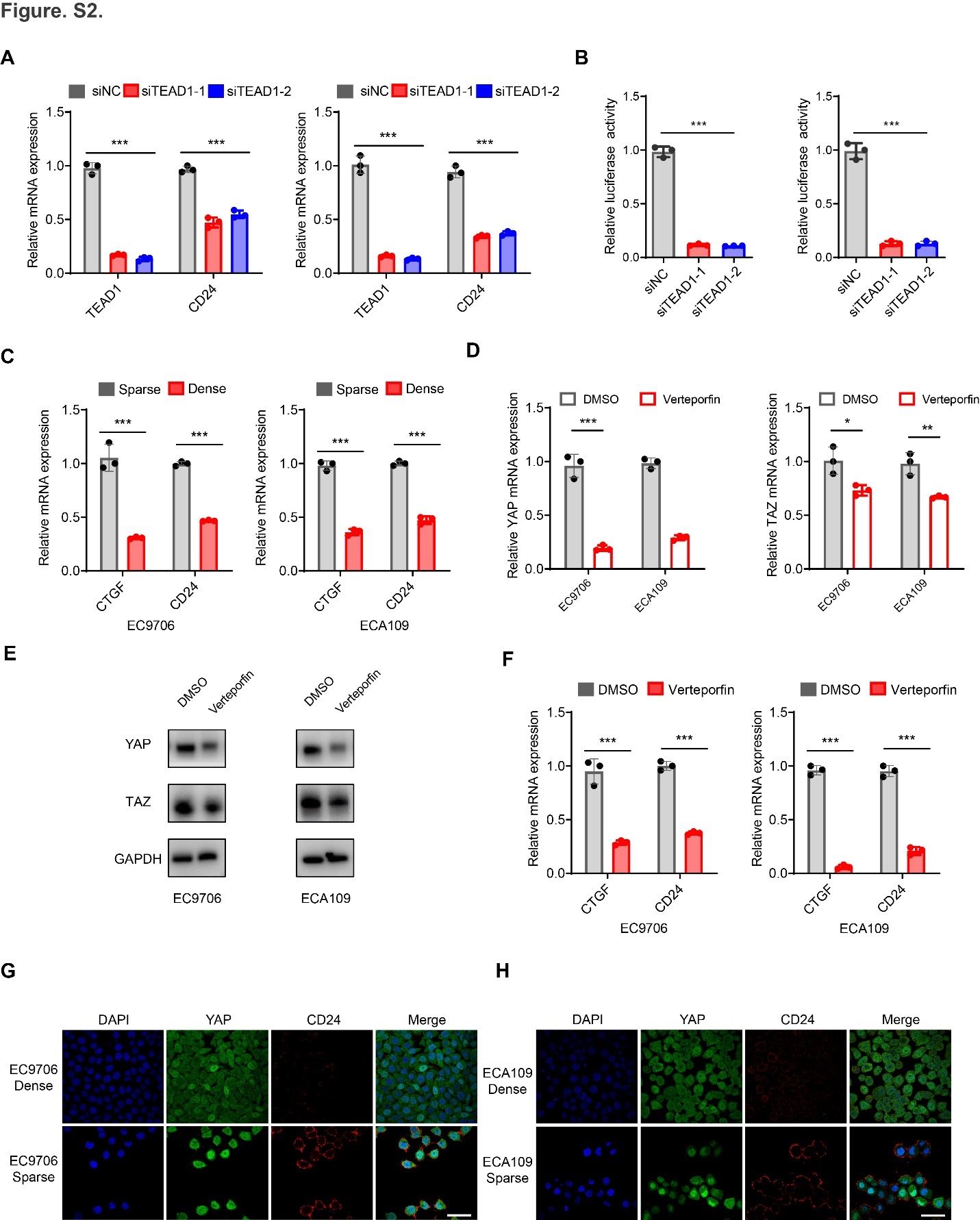


**Figure. S2.**

**CD24 expression is regulated by Hippo signaling pathway**

(A) The expression level of CD24 was significantly down-regulated after silencing TEAD1.

(B) The CD24 promoter activity was significantly down-regulated after silencing TEAD1.

(C) High cell density inhibited CD24 expression in different human esophageal squamous cells.

(D) qRT-PCR analysis of YAP and TAZ mRNA levels in ESCC cells treated with DMSO or VP (2.5 mM) for 24 hours.

(E) Western blot analysis of YAP and TAZ expression after ESCC cells treated with DMSO or VP (2.5 mM) for 24 hours.

(F) qRT-PCR analysis of CD24 mRNA levels in ESCC cells treated with DMSO or VP (2.5 mM) for 24 hours.

(G-H) High-cell density inhibited nuclear localization of YAP and reduced the level of membrane CD24 expression, scale bars, 100 μm.


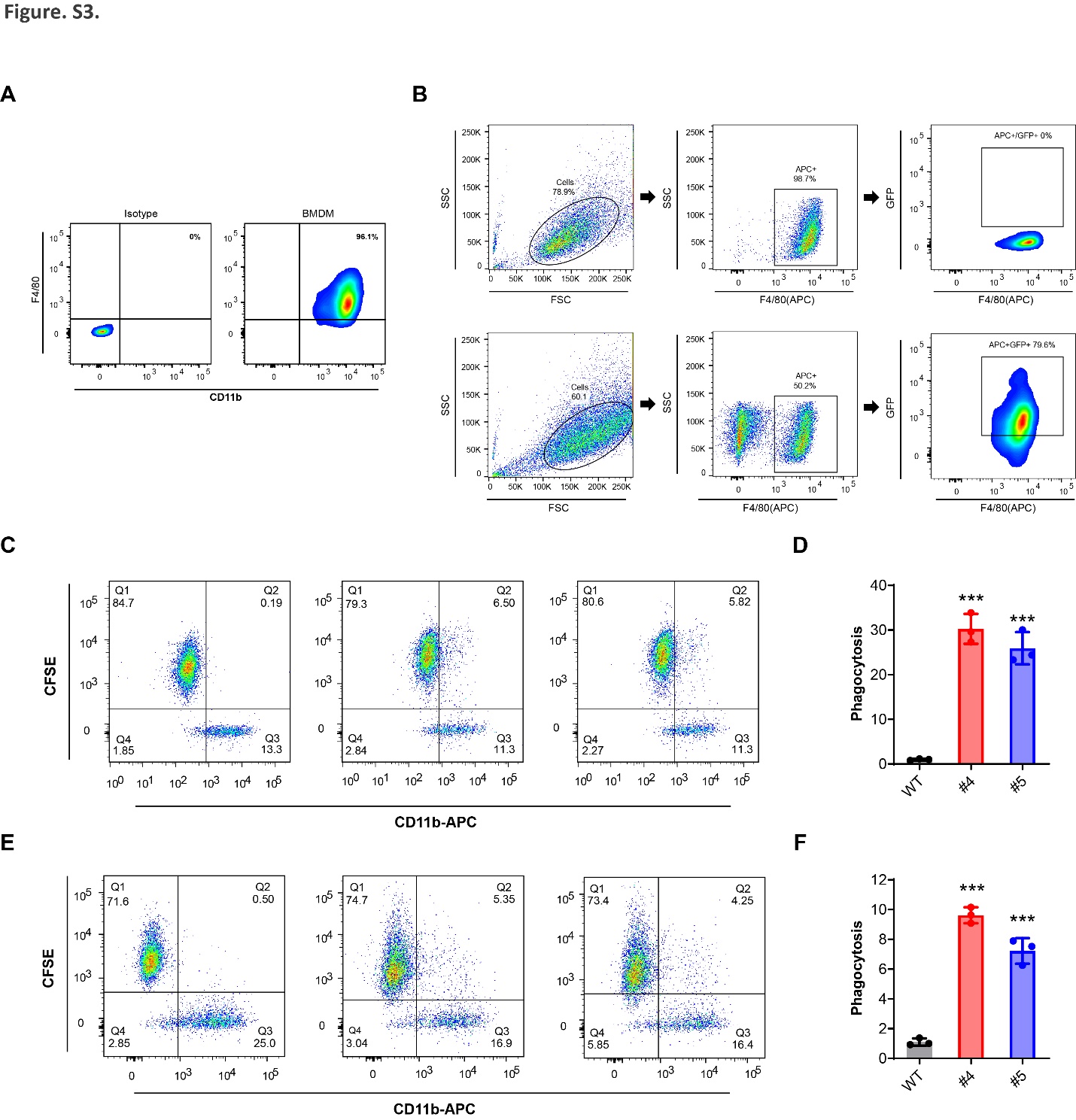


**Figure. S3.**

**Gating strategy for phagocytosis assay**

1. Mature BMDMs were defined as CD11b + F4/80 + subpopulations.
2. Schematic view of the gating strategy used for a flow cytometry-based phagocytosis assay analysis.

(C-F) Human peripheral blood-derived macrophages exhibit significantly higher phagocytosis rate towards YAP knockout ESCC cells compared to the wild-type. The phagocytosis rate calculation formula is CD11b^+^CFSE^+^(Q2) / CFSE^+^(Q1+Q2) × 100%, *** *P*＜0.001 vs WT.


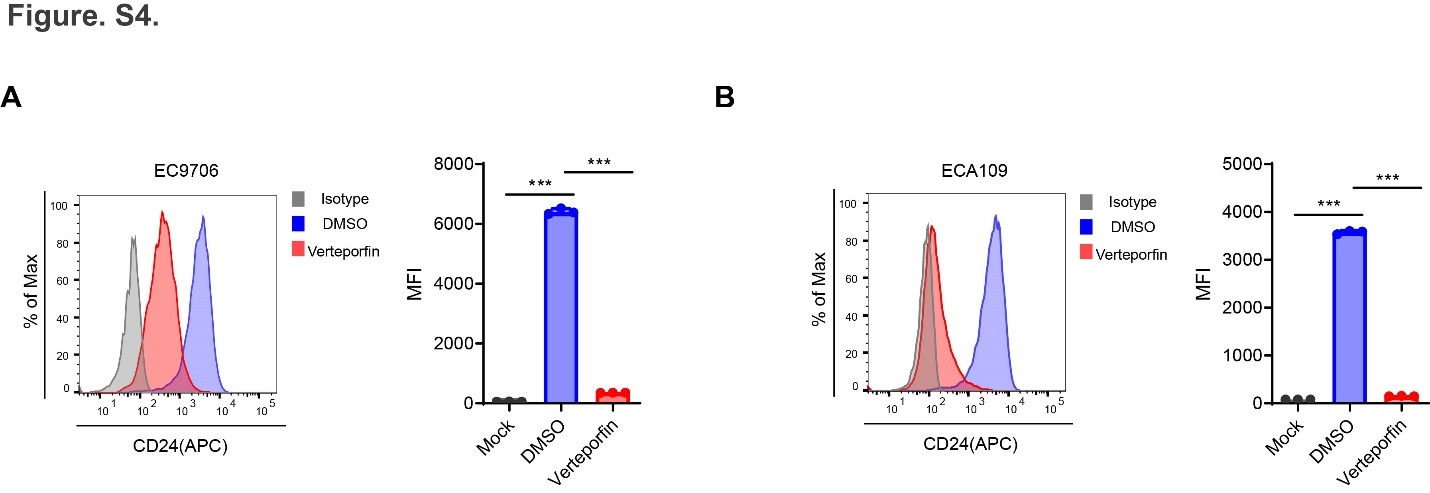


**Figure. S4.**

**VP treatment inhibited membrane CD24 expression in ESCC**

(A-B) Flow cytometry results showed that the CD24 membrane expression in EC9706 (A) and ECA109 (B) decreased after VP treatment.


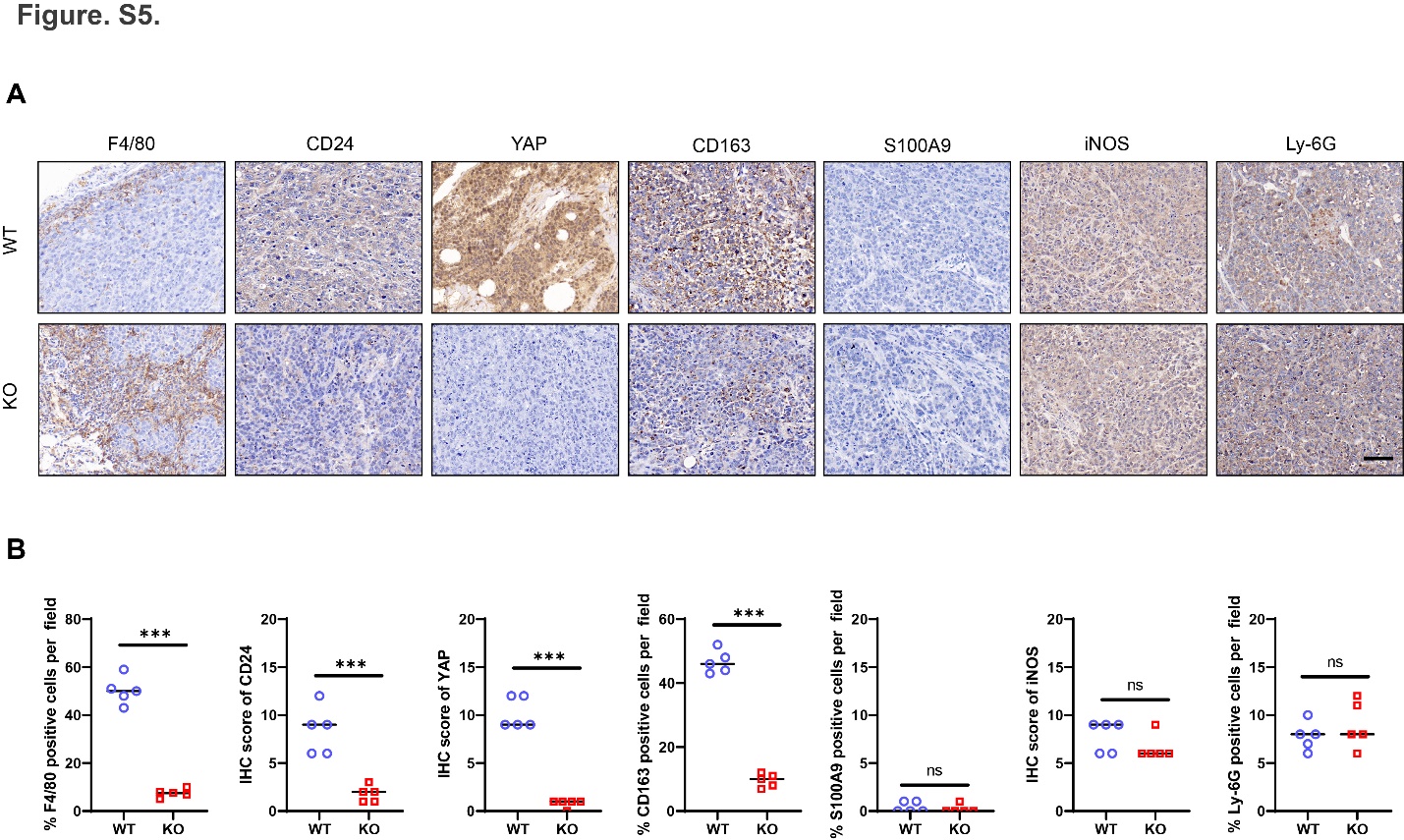


**Figure. S5.**

**Immunohistochemical analysis of xenograft tumor tissues.**

(A) Immunohistochemical analysis of F4/80, CD24, YAP, CD163, S100A9, iNOS, and Ly-6G expression levels in xenograft tumors. Scale bars, 50 μm.

(B) Immunohistochemical analysis and quantification (n = 5 in each group).


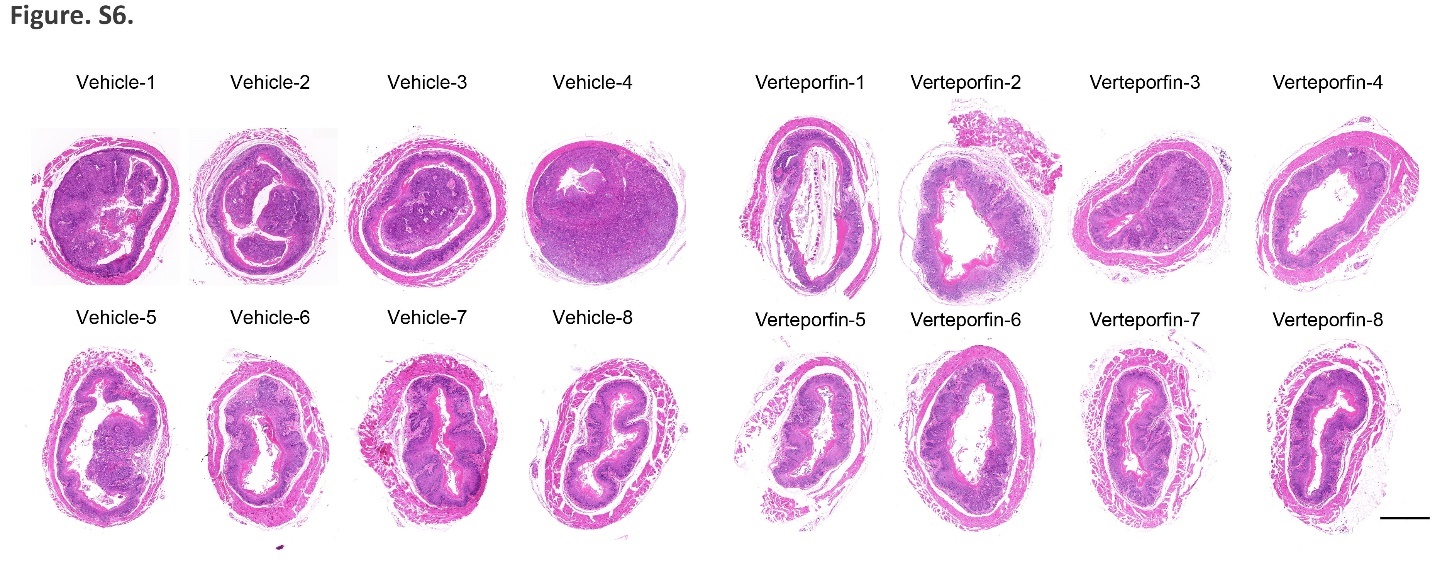


**Figure. S6.**

**Pathological features of both the control and verteporfin–treated mice after administration of eight doses of VP. Scale bars, 500μm**


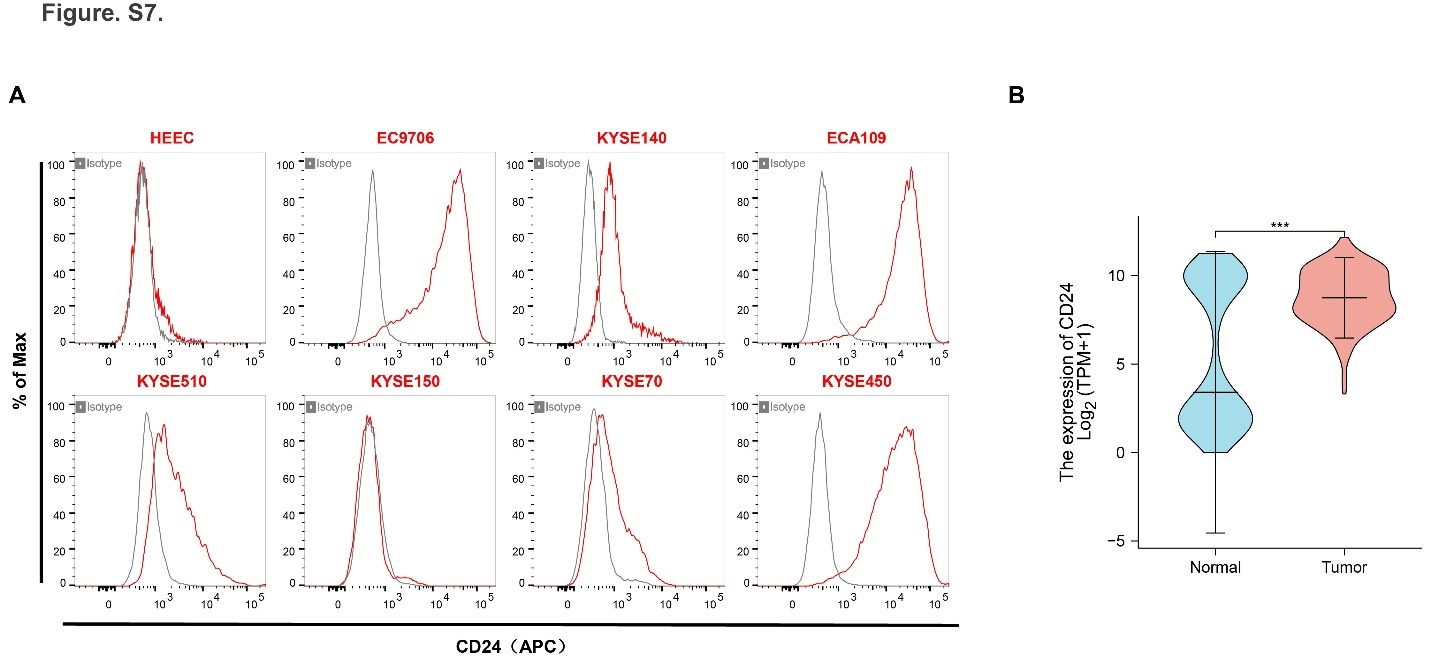


**Figure. S7.**

**CD24 is highly expressed in ESCC samples and cell lines**

(A) The expression level of CD24 is increased in ESCC cell lines compared to that of normal esophageal epithelial cell lines (HEEC).

(B) The data of TCGA showed a high expression of CD24 in esophageal cancer.


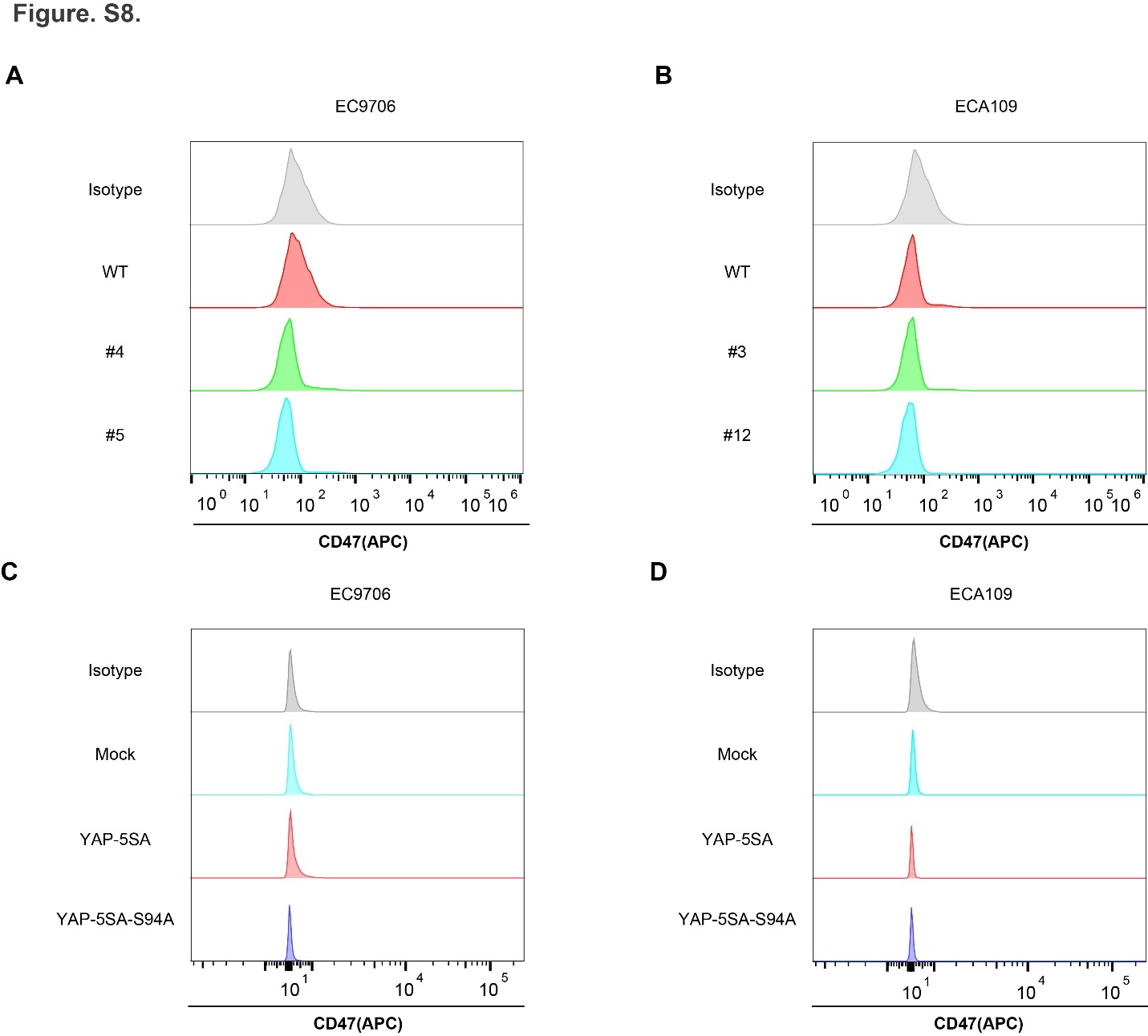


**Figure. S8.**

**CD47 expression was not associated with YAP depletion or activation**

(A-B) YAP knockout does not affect the expression of CD47 in ESCC cells.

(C-D) YAP activation does not affect the expression of CD47 in ESCC cells.


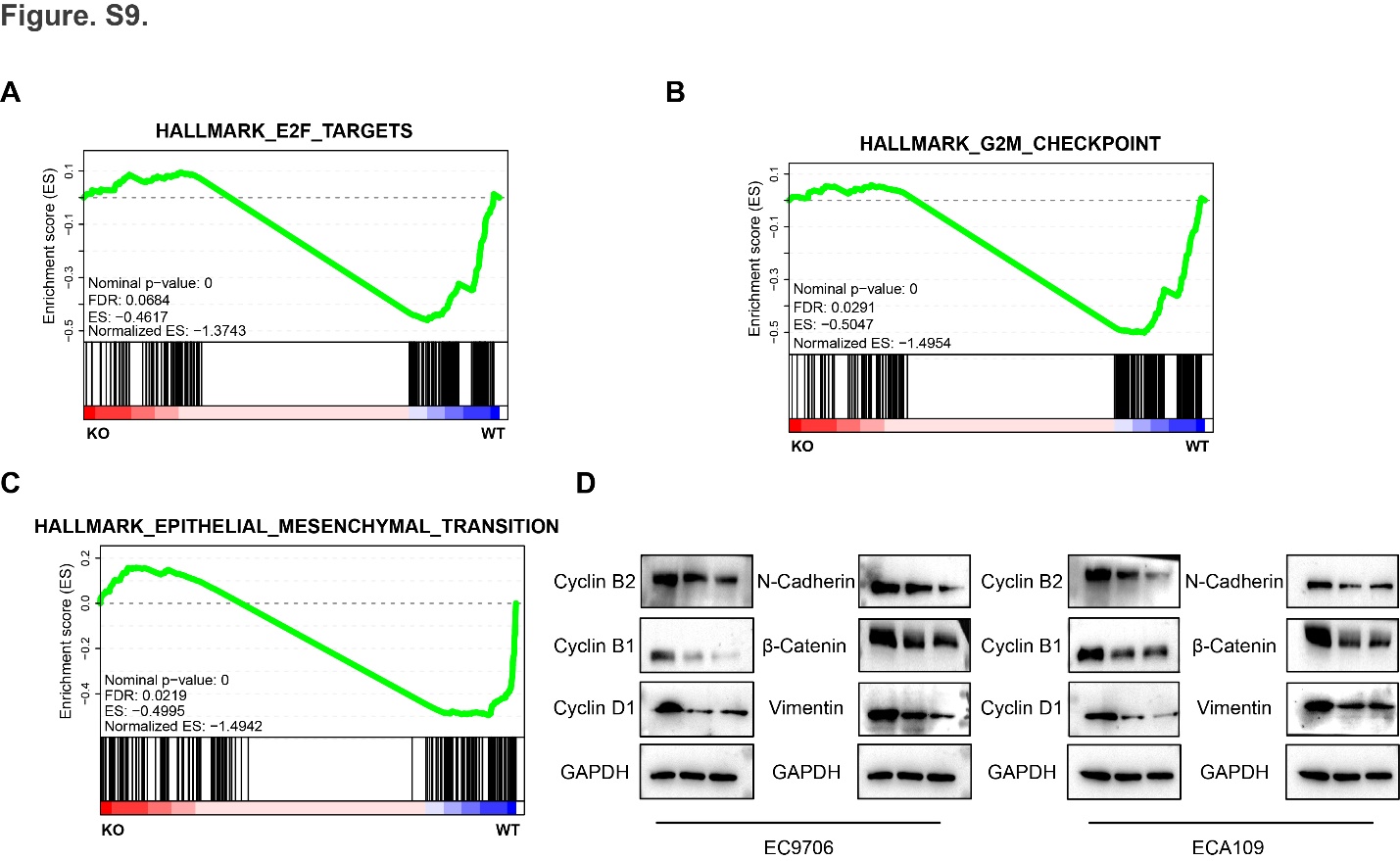


**Figure. S9.**

**GSEA analysis of RNA-seq data and validation by Western blot.**

(A-C) GSEA analysis of RNA-seq data indicates a significant enrichment of E2F target genes, G2M checkpoint genes and Epithelial–Mesenchymal Transition genes in WT cells after YAP knockout.

(D) WB validation of candidate molecules.


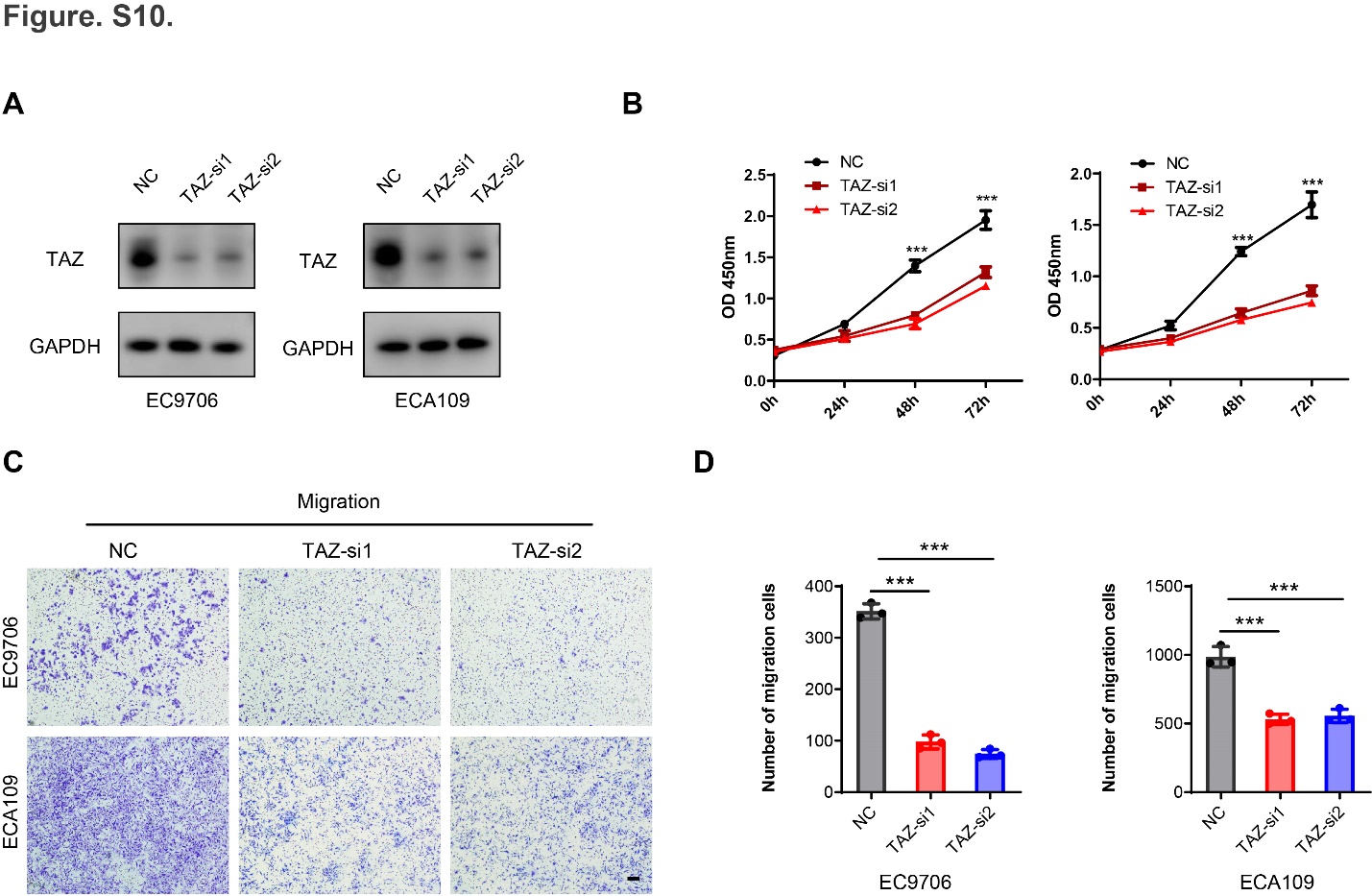


**Figure. S10.**

**Knockdown of TAZ affects the proliferation and migration of ESCC cells.**

1. WB of TAZ knockdown in ESCC cells.

(B-C) Knockdown of TAZ affects the proliferation and migration of esophageal squamous cell carcinoma cells. Scale bars, 100 μm.

(D) Statistical analysis for the number of migration cells.

**Supplementary Table1**

**Sequences of the siRNA**

| Name | species | Forward (5' -> 3') | Reverse (5' -> 3') |
| --- | --- | --- | --- |
| siTEAD1-1 | human | GUGUGUUUGAAGUUUCAAATT | UUUGAAACUUCAAACACACTT |
| siTEAD1-2 | human | CGAUUUGUAUACCGAAUAATT | UUAUUCGGUAUACAAAUCGTT |
| si-NC | human | UUCUCCGAACGUGUCACGUTT | ACGUGACACGUUCGGAGAATT |

**Supplementary Table2**

**Clinicopathological correlation of YAP and CD24 expression in ESCC**


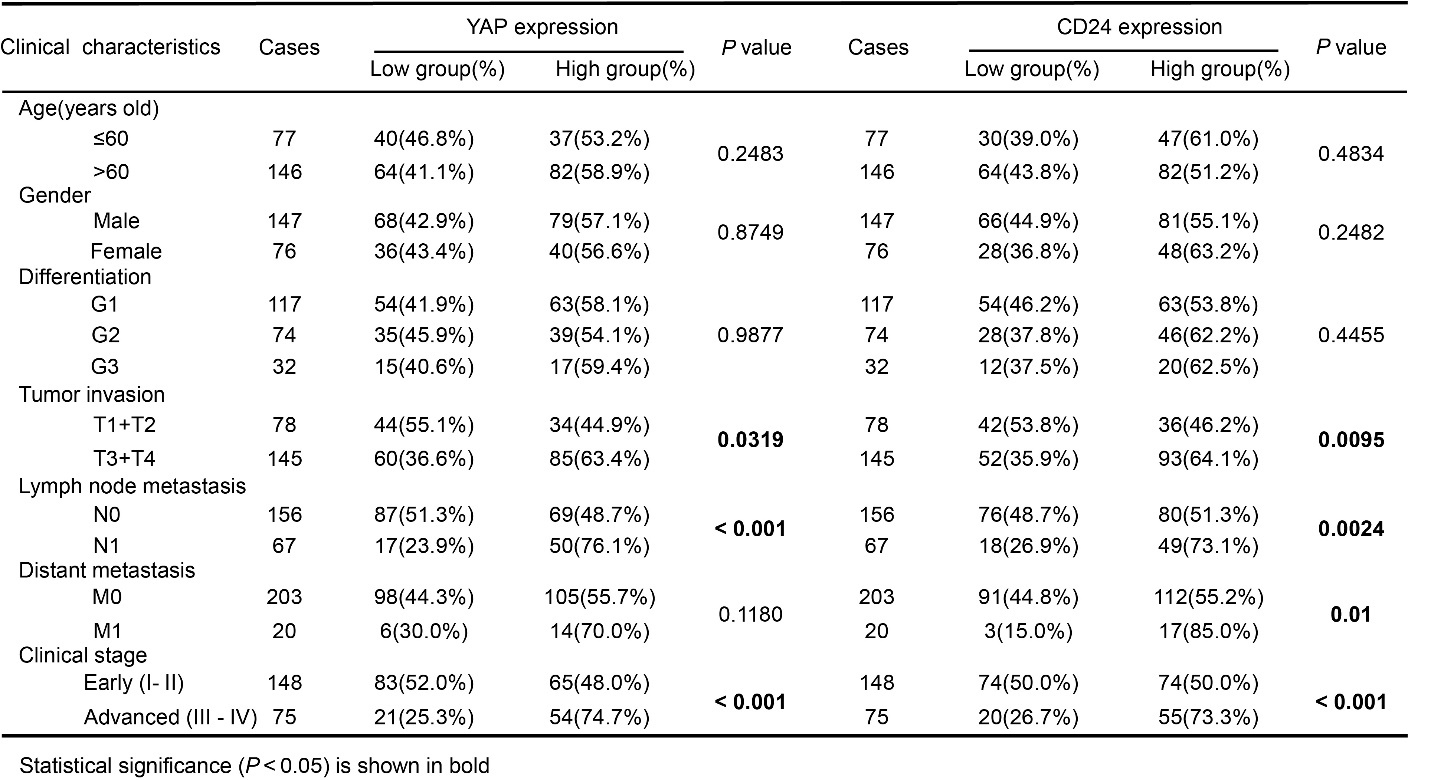


**Supplementary Table3**

**Clinicopathological correlation of CD68 expression in ESCC**


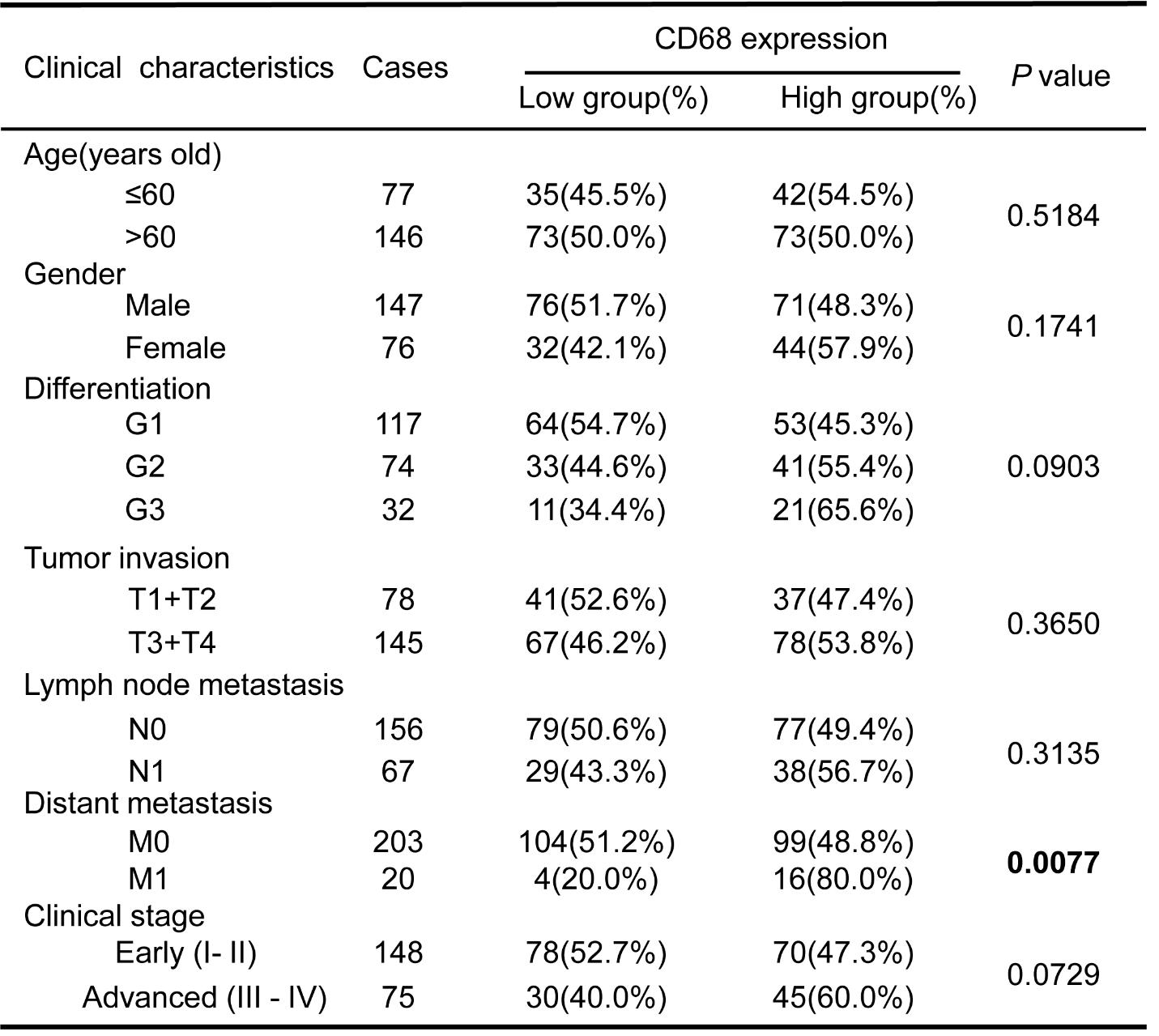


**Supplementary Table4**

**The guide RNA sequence and screening primers**

| Oligos | Forward (5' -> 3') | Reverse (5' -> 3') |
| --- | --- | --- |
| pX460-YAP-KO-1 | CACCGTAATAGGCCAGTACTGATGC | AAACGCATCAGTACTGGCCTATTAC |
| pX461-YAP-KO-2 | CACCGACCCCCACTGGAGTAGTCTC | AAACGAGACTACTCCAGTGGGGGTC |
| pX460-YAP-KO-3 | CACCGTGTACCTCTGCCAGCAGGTT | AAACAACCTGCTGGCAGAGGTACAC |
| screening primers | GCAATTAAGCGCTGACTGGG | GGCTCAGAACCAAGACTCAGTAA |

**Supplementary Table5**

**Primer sequences for Real-time PCR**

| Gene | species | Forward (5' -> 3') | Reverse (5' -> 3') |
| --- | --- | --- | --- |
| YAP | human | TCCACCAGTGCAGCAGAATA | TTGGGTCTAGCCAAGAGGTG |
| TEAD1 | human | AACTCAGGACAGGCAAGACG | AGCTTGGAATGAAAATCACGA |
| CTGF | human | GCACAAGGGCCTATTCTGTC | ACGTGCACTGGTACTTGCAG |
| CD24 | human | CTGCTGGCACTGCTCCTAC | ACCACGAAGAGACTGGCTGT |
| GAPDH | human | CGAGATCCCTCCAAAATCAA | TTCACACCCATGACGAACAT |

**Supplementary Table6**

**Primer sequences for ChIP-qPCR**

| Oligos | Forward (5' -> 3') | Reverse (5' -> 3') |
| --- | --- | --- |
| CD24-site1 | TCTCGAACGGTTCTGCTCTT | GGCGGTCCCACTACAATTCA |
| CD24-site2 | AAGCAGCTTGTGGAGGACA | GCCCAGCTTCTCCATTCCTT |

**Supplementary Table7**

**Clinical characteristics of 223 human ESCC tissue samples**

| No. | Age | Sex | Pathology diagnosis | Clinical stages | Grade | TNM | Date of death |
| --- | --- | --- | --- | --- | --- | --- | --- |
| 201401772 | 66 | M | ESCC | Ⅲ | G1 | T3N1M0 | 2016/4/17 |
| 201402586 | 60 | F | ESCC | Ⅲ | G2 | T3N2M0 | 2016/11/11 |
| 201402742 | 57 | M | ESCC | Ⅱ | G1 | T2N0M0 | survival |
| 201402843 | 68 | F | ESCC | Ⅲ | G1 | T3N1M0 | 2015/1/10 |
| 201403038 | 57 | M | ESCC | Ⅱ | G2 | T2N0M0 | survival |
| 201403091 | 57 | F | ESCC | Ⅲ | G1 | T3N1M0 | 2014/12/21 |
| 201403147 | 64 | F | ESCC | Ⅰ | G1 | T1BN0M0 | survival |
| 201403182 | 70 | M | ESCC | Ⅱ | G1 | T2N0M0 | survival |
| 201403183 | 67 | F | ESCC | Ⅳ-B | G2 | T2N1M1 | 2014/7/18 |
| 201403235 | 52 | M | ESCC | Ⅳ-A | G1 | T3N3M0 | 2018/4/24 |
| 201403365 | 40 | F | ESCC | Ⅳ-B | G3 | T3N1M1 | 2015/5/27 |
| 201403368 | 56 | F | ESCC | Ⅳ-B | G1 | T3N1M1 | 2015/2/27 |
| 201403464 | 68 | M | ESCC | Ⅰ | G3 | T1BN0M0 | survival |
| 201403512 | 61 | F | ESCC | Ⅱ | G1 | T2N0M0 | 2018/11/1 |
| 201403592 | 74 | F | ESCC | Ⅱ | G2 | T2N0M0 | 2015/8/1 |
| 201403902 | 65 | M | ESCC | Ⅰ | G1 | T1BN0M0 | survival |
| 201403906 | 61 | M | ESCC | Ⅳ-A | G1 | T4AN0M0 | survival |
| 201404002 | 51 | F | ESCC | Ⅱ | G2 | T3N0M0 | survival |
| 201404060 | 71 | M | ESCC | Ⅲ | G1 | T3N1M0 | 2019/1/11 |
| 201404106 | 54 | F | ESCC | Ⅳ-A | G1 | T4AN0M0 | survival |
| 201404193 | 63 | M | ESCC | Ⅰ | G1 | T1BN0M0 | survival |
| 201404297 | 74 | F | ESCC | Ⅳ-B | G3 | T4AN2M1 | 2017/3/21 |
| 201404298 | 66 | M | ESCC | Ⅱ | G3 | T2N0M0 | survival |
| 201404364 | 66 | M | ESCC | Ⅳ-A | G1 | T4AN0M0 | 2016/8/15 |
| 201404391 | 67 | M | ESCC | Ⅱ | G1 | T2N0M0 | survival |
| 201404500 | 57 | M | ESCC | Ⅱ | G1 | T3N0M0 | survival |
| 201404501 | 71 | F | ESCC | Ⅰ | G2 | T1BN0M0 | survival |
| 201404523 | 67 | M | ESCC | Ⅱ | G1 | T3N0M0 | 2018/11/28 |
| 201404658 | 66 | M | ESCC | Ⅱ | G2 | T3N0M0 | 2015/9/18 |
| 201404721 | 59 | M | ESCC | Ⅱ | G2 | T2N0M0 | 2015/4/30 |
| 201405057 | 67 | F | ESCC | Ⅰ | G2 | T1BN0M0 | survival |
| 201405214 | 60 | M | ESCC | Ⅱ | G3 | T2N0M0 | 2015/4/9 |
| 201405296 | 77 | M | ESCC | Ⅱ | G2 | T3N0M0 | 2014/8/14 |
| 201405532 | 70 | M | ESCC | Ⅱ | G2 | T3N0M0 | survival |
| 201406128 | 44 | M | ESCC | Ⅳ-B | G2 | T3N0M1 | 2016/4/6 |
| 201406276 | 54 | F | ESCC | Ⅱ | G2 | T3N0M0 | 2014/8/6 |
| 201406329 | 61 | M | ESCC | Ⅱ | G1 | T2N0M0 | 2016/5/11 |
| 201406373 | 52 | M | ESCC | Ⅳ-B | G1 | T3N0M1 | 2015/10/13 |
| 201406924 | 63 | F | ESCC | Ⅰ | G1 | T1BN0M0 | survival |
| 201407091 | 69 | M | ESCC | Ⅱ | G2 | T3N0M0 | survival |
| 201407338 | 57 | M | ESCC | Ⅱ | G1 | T3N0M0 | survival |
| 201407489 | 61 | M | ESCC | Ⅳ-B | G3 | T3N0M1 | 2015/1/9 |
| 201407683 | 56 | M | ESCC | Ⅳ-A | G1 | T4AN0M0 | 2014/9/14 |
| 201407707 | 62 | M | ESCC | Ⅱ | G1 | T2N0M0 | survival |
| 201407800 | 74 | F | ESCC | Ⅱ | G1 | T2N0M0 | survival |
| 201407825 | 59 | F | ESCC | Ⅳ-B | G1 | T2N0M1 | 2016/7/24 |
| 201407825 | 72 | M | ESCC | Ⅱ | G3 | T3N0M0 | survival |
| 201407958 | 56 | F | ESCC | Ⅱ | G2 | T2N1M0 | 2017/4/18 |
| 201408091 | 58 | M | ESCC | Ⅱ | G2 | T2NIM0 | survival |
| 201408284 | 62 | M | ESCC | Ⅳ-B | G2 | T4BN1M1 | 2017/2/29 |
| 201408343 | 59 | M | ESCC | Ⅳ-A | G1 | T4AN1M0 | survival |
| 201408344 | 64 | F | ESCC | Ⅱ | G1 | T2N0M0 | survival |
| 201408399 | 58 | F | ESCC | Ⅱ | G1 | T3N0M0 | survival |
| 201408519 | 78 | M | ESCC | Ⅱ | G2 | T2N1M0 | survival |
| 201408941 | 60 | F | ESCC | Ⅰ | G2 | T2N0M0 | survival |
| 201408966 | 73 | M | ESCC | Ⅲ | G2 | T3N1M0 | 2015/6/14 |
| 201408998 | 71 | M | ESCC | Ⅳ-A | G1 | T4AN0M0 | survival |
| 201409211 | 59 | F | ESCC | Ⅳ-B | G3 | T1BN0M1 | 2015/2/8 |
| 201409248 | 58 | F | ESCC | Ⅱ | G2 | T2N0M0 | 2017/3/20 |
| 201409280 | 67 | M | ESCC | Ⅱ | G3 | T3N0M0 | 2018/11/21 |
| 201409536 | 61 | F | ESCC | Ⅱ | G1 | T3N0M0 | survival |
| 201409570 | 59 | M | ESCC | Ⅰ | G2 | T1BN0M0 | survival |
| 201409637 | 72 | F | ESCC | Ⅱ | G2 | T2N0M0 | 2015/3/26 |
| 201409930 | 67 | M | ESCC | Ⅳ-B | G1 | T3N1M1 | 2015/6/10 |
| 201410018 | 75 | F | ESCC | Ⅳ-B | G2 | T3N1M1 | 2014/12/12 |
| 201410163 | 65 | F | ESCC | Ⅱ | G1 | T3N0M0 | survival |
| 201410336 | 76 | M | ESCC | Ⅲ | G2 | T3N1M0 | 2015/7/22 |
| 201410501 | 58 | M | ESCC | Ⅱ | G1 | T2N0M0 | survival |
| 201410528 | 59 | F | ESCC | Ⅱ | G2 | T2N0M0 | 2017/5/26 |
| 201410798 | 64 | M | ESCC | Ⅰ | G3 | T1BN0M0 | 2017/5/5 |
| 201410867 | 63 | M | ESCC | Ⅱ | G2 | T3N0M0 | survival |
| 201410985 | 51 | M | ESCC | Ⅲ | G3 | T3N2M0 | 2015/9/11 |
| 201411004 | 75 | M | ESCC | Ⅳ-B | G1 | T3N1M1 | 2016/9/13 |
| 201411078 | 60 | M | ESCC | Ⅲ | G2 | T3N2M0 | 2016/7/14 |
| 201411130 | 60 | M | ESCC | Ⅱ | G1 | T2N0M0 | survival |
| 201411202 | 63 | M | ESCC | Ⅱ | G1 | T3N0M0 | survival |
| 201411242 | 66 | M | ESCC | Ⅱ | G3 | T3N0M0 | survival |
| 201411609 | 60 | M | ESCC | Ⅲ | G1 | T3N1M0 | 2015/6/24 |
| 201411768 | 62 | M | ESCC | Ⅱ | G2 | T2N0M0 | 2014/11/19 |
| 201411795 | 65 | F | ESCC | Ⅱ | G1 | T3N0M0 | 2017/3/31 |
| 201411808 | 55 | M | ESCC | Ⅱ | G1 | T3N0M0 | 2019/7/5 |
| 201411913 | 66 | F | ESCC | Ⅲ | G2 | T3N2M0 | 2015/12/5 |
| 201411936 | 65 | M | ESCC | Ⅳ-B | G1 | T3NIM1 | 2016/7/16 |
| 201412055 | 72 | F | ESCC | Ⅱ | G1 | T3N0M0 | 2015/6/10 |
| 201412056 | 57 | M | ESCC | Ⅲ | G1 | T3N1M0 | survival |
| 201412079 | 59 | M | ESCC | Ⅱ | G1 | T3N0M0 | 2015/4/11 |
| 201412167 | 65 | M | ESCC | Ⅱ | G1 | T3N0M0 | survival |
| 201412203 | 51 | M | ESCC | Ⅱ | G1 | T3N0M0 | 2018/11/11 |
| 201412934 | 73 | F | ESCC | Ⅲ | G1 | T3N2M0 | 2016/6/1 |
| 201413161 | 75 | F | ESCC | Ⅲ | G1 | T2N2M0 | 2015/8/8 |
| 201413187 | 63 | M | ESCC | Ⅱ | G2 | T3N0M0 | survival |
| 201413311 | 60 | F | ESCC | Ⅱ | G1 | T3N0M0 | survival |
| 201413312 | 47 | M | ESCC | Ⅳ-B | G3 | T3N3M1 | 2015/7/15 |
| 201413313 | 65 | M | ESCC | Ⅱ | G2 | T2N1M0 | 2015/3/15 |
| 201413404 | 64 | F | ESCC | Ⅱ | G1 | T2N0M0 | survival |
| 201413428 | 57 | F | ESCC | Ⅲ | G2 | T3N2M0 | 2018/10/18 |
| 201413490 | 72 | M | ESCC | Ⅳ-B | G3 | T2N0M1 | 2016/6/18 |
| 201413509 | 66 | F | ESCC | Ⅱ | G1 | T3N0M0 | 2016/6/18 |
| 201413563 | 63 | M | ESCC | Ⅱ | G1 | T2N0M0 | 2017/11/22 |
| 201413564 | 75 | M | ESCC | Ⅰ | G1 | T1BN0M0 | survival |
| 201500107 | 58 | F | ESCC | Ⅱ | G1 | T3N0M0 | survival |
| 201500191 | 68 | M | ESCC | Ⅱ | G1 | T3N0M0 | survival |
| 201500227 | 62 | F | ESCC | Ⅱ | G2 | T3N0M0 | survival |
| 201500376 | 70 | M | ESCC | Ⅱ | G2 | T2N0M0 | 2019/3/9 |
| 201500504 | 70 | M | ESCC | Ⅲ | G1 | T3N1M0 | 2016/3/8 |
| 201500965 | 61 | M | ESCC | Ⅲ | G2 | T2N2M0 | 2016/7/15 |
| 201501160 | 70 | M | ESCC | Ⅱ | G3 | T2N0M0 | survival |
| 201501289 | 65 | M | ESCC | Ⅱ | G3 | T3N0M0 | survival |
| 201501538 | 73 | M | ESCC | Ⅲ | G1 | T3N1M0 | 2015/7/7 |
| 201501597 | 57 | F | ESCC | Ⅲ | G1 | T3N1M0 | survival |
| 201501621 | 65 | F | ESCC | Ⅰ | G2 | T1BN0M0 | survival |
| 201501704 | 58 | M | ESCC | Ⅲ | G1 | T3N2M0 | 2016/2/14 |
| 201501717 | 64 | M | ESCC | Ⅱ | G1 | T2N0M0 | 2016/2/14 |
| 201502248 | 71 | F | ESCC | Ⅱ | G2 | T3N0M0 | survival |
| 201502282 | 58 | M | ESCC | Ⅱ | G2 | T3N0M0 | survival |
| 201502283 | 64 | M | ESCC | Ⅱ | G1 | T3N0M0 | survival |
| 201502284 | 75 | F | ESCC | Ⅲ | G2 | T3N1M0 | 2016/5/3 |
| 201502405 | 65 | M | ESCC | Ⅱ | G1 | T3N0M0 | survival |
| 201502436 | 62 | M | ESCC | Ⅱ | G1 | T3N0M0 | 2016/6/5 |
| 201502588 | 66 | M | ESCC | Ⅱ | G2 | T2N0M0 | 2017/7/6 |
| 201502786 | 64 | F | ESCC | Ⅲ | G1 | T3N1M0 | 2017/10/9 |
| 201502814 | 72 | F | ESCC | Ⅲ | G3 | T3N2M0 | 2016/5/13 |
| 201502913 | 62 | F | ESCC | Ⅲ | G1 | T3N1M0 | survival |
| 201502990 | 58 | M | ESCC | Ⅱ | G1 | T2N0M0 | 2016/3/12 |
| 201503241 | 57 | M | ESCC | Ⅱ | G3 | T3N0M0 | 2015/7/25 |
| 201503280 | 67 | M | ESCC | Ⅱ | G1 | T3N0M0 | 2017/2/20 |
| 201503686 | 60 | M | ESCC | Ⅱ | G2 | T3N0M0 | survival |
| 201504330 | 62 | M | ESCC | Ⅱ | G1 | T2N0M0 | 2016/4/17 |
| 201504390 | 59 | M | ESCC | Ⅱ | G2 | T3N0M0 | survival |
| 201504438 | 76 | M | ESCC | Ⅱ | G3 | T3N0M0 | 2015/8/21 |
| 201504517 | 57 | F | ESCC | Ⅱ | G2 | T3N0M0 | survival |
| 201504575 | 62 | M | ESCC | Ⅱ | G3 | T3N0M0 | 2016/1/25 |
| 201504644 | 69 | M | ESCC | Ⅱ | G1 | T3N0M0 | survival |
| 201504927 | 68 | M | ESCC | Ⅱ | G1 | T2N0M0 | 2017/4/4 |
| 201505086 | 69 | F | ESCC | Ⅳ-B | G1 | T3N1M1 | 2016/6/5 |
| 201505265 | 57 | M | ESCC | Ⅲ | G2 | T3N2M0 | 2016/5/7 |
| 201505383 | 81 | F | ESCC | Ⅲ | G1 | T3N1M0 | 2015//7/11 |
| 201505465 | 74 | F | ESCC | Ⅱ | G2 | T3N0M0 | 2017/4/15 |
| 201505504 | 63 | F | ESCC | Ⅱ | G1 | T3N0M0 | survival |
| 201505748 | 60 | M | ESCC | Ⅱ | G2 | T2N0M0 | survival |
| 201505945 | 62 | M | ESCC | Ⅱ | G1 | T3N0M0 | 2016/6/14 |
| 201505977 | 73 | M | ESCC | Ⅱ | G1 | T2N0M0 | survival |
| 201506186 | 59 | M | ESCC | Ⅲ | G2 | T3N1M0 | 2016/7/1 |
| 201506339 | 67 | F | ESCC | Ⅱ | G2 | T2N0M0 | survival |
| 201506340 | 69 | M | ESCC | Ⅱ | G1 | T3N0M0 | survival |
| 201506517 | 61 | F | ESCC | Ⅱ | G2 | T3N0M0 | survival |
| 201506538 | 65 | M | ESCC | Ⅱ | G1 | T3N0M0 | survival |
| 201506757 | 63 | M | ESCC | Ⅱ | G1 | T2N0M0 | survival |
| 201507122 | 59 | M | ESCC | Ⅱ | G2 | T2N0M0 | survival |
| 201507123 | 63 | M | ESCC | Ⅳ-B | G2 | T3N2M1 | 2016/2/19 |
| 201507549 | 61 | M | ESCC | Ⅱ | G1 | T3N0M0 | survival |
| 201507654 | 62 | M | ESCC | Ⅲ | G1 | T3N1M0 | survival |
| 201507862 | 48 | M | ESCC | Ⅱ | G2 | T3N0M0 | 2018/11/14 |
| 201507863 | 75 | M | ESCC | Ⅰ | G1 | T1BN0M0 | 2017/5/13 |
| 201508011 | 62 | M | ESCC | Ⅱ | G3 | T3N0M0 | survival |
| 201508012 | 68 | M | ESCC | Ⅲ | G1 | T3N2M0 | 2016/10/16 |
| 201508028 | 63 | M | ESCC | Ⅰ | G1 | T1BN0M0 | survival |
| 201508319 | 66 | M | ESCC | Ⅱ | G2 | T3N0M0 | survival |
| 201508321 | 64 | F | ESCC | Ⅱ | G2 | T2N0M0 | survival |
| 201508358 | 58 | F | ESCC | Ⅱ | G2 | T3N0M0 | survival |
| 201508478 | 66 | M | ESCC | Ⅱ | G1 | T3N0M0 | survival |
| 201508479 | 71 | M | ESCC | Ⅱ | G1 | T3N0M0 | 2015/12/27 |
| 201508532 | 68 | M | ESCC | Ⅱ | G2 | T2N0M0 | survival |
| 201508604 | 60 | M | ESCC | Ⅱ | G1 | T3N0M0 | 2016/7/3 |
| 201508686 | 59 | F | ESCC | Ⅱ | G2 | T3N0M0 | 2017/9/27 |
| 201508687 | 58 | M | ESCC | Ⅱ | G1 | T3N0M0 | survival |
| 201508789 | 62 | M | ESCC | Ⅱ | G2 | T3N0M0 | 2018/2/27 |
| 201508790 | 75 | F | ESCC | Ⅱ | G1 | T2N0M0 | survival |
| 201508887 | 73 | M | ESCC | Ⅱ | G1 | T3N0M0 | 2017/10/14 |
| 201509200 | 61 | M | ESCC | Ⅲ | G3 | T3N1M0 | 2017/6/17 |
| 201509279 | 71 | M | ESCC | Ⅱ | G1 | T3N0M0 | 2016/9/19 |
| 201509338 | 64 | M | ESCC | Ⅲ | G2 | T3N2M0 | 2018/12/20 |
| 201509595 | 62 | M | ESCC | Ⅲ | G1 | T3N1M0 | survival |
| 201510098 | 63 | M | ESCC | Ⅰ | G2 | T1BN0M0 | survival |
| 201510099 | 65 | M | ESCC | Ⅱ | G1 | T3N0M0 | 2018/1/11 |
| 201510100 | 57 | M | ESCC | Ⅲ | G2 | T3N1M0 | survival |
| 201510149 | 64 | F | ESCC | Ⅱ | G1 | T2N0M0 | survival |
| 201510163 | 60 | F | ESCC | Ⅲ | G3 | T3N1M0 | 2016/3/11 |
| 201510214 | 59 | F | ESCC | Ⅱ | G2 | T3N0M0 | 2017/7/12 |
| 201510243 | 58 | M | ESCC | Ⅰ | G2 | T1AN0M0 | survival |
| 201510280 | 52 | M | ESCC | Ⅱ | G1 | T3N0M0 | survival |
| 201510336 | 59 | F | ESCC | Ⅱ | G1 | T2N0M0 | survival |
| 201510355 | 63 | M | ESCC | Ⅲ | G1 | T3N1M0 | 2018/11/17 |
| 201510519 | 75 | F | ESCC | Ⅱ | G3 | T3N0M0 | survival |
| 201510806 | 67 | M | ESCC | Ⅱ | G2 | T3N0M0 | 2016/10/7 |
| 201510870 | 71 | F | ESCC | Ⅱ | G1 | T2N0M0 | survival |
| 201510932 | 66 | M | ESCC | Ⅱ | G1 | T3N0M0 | survival |
| 201510935 | 65 | F | ESCC | Ⅱ | G1 | T3N0M0 | survival |
| 201511060 | 62 | M | ESCC | Ⅱ | G1 | T3N0M0 | survival |
| 201511106 | 55 | M | ESCC | Ⅱ | G3 | T2N0M0 | survival |
| 201511148 | 66 | F | ESCC | Ⅰ | G1 | T1BN0M0 | survival |
| 201511185 | 30 | M | ESCC | Ⅱ | G3 | T2N0M0 | 2016/6/15 |
| 201511325 | 65 | F | ESCC | Ⅲ | G2 | T3N1M0 | 2016/5/15 |
| 201511647 | 58 | M | ESCC | Ⅱ | G2 | T3N0M0 | 2018/4/26 |
| 201511648 | 66 | F | ESCC | Ⅲ | G2 | T3N1M0 | survival |
| 201511758 | 58 | M | ESCC | Ⅳ-B | G1 | T3N1M1 | 2017/11/30 |
| 201511780 | 58 | M | ESCC | Ⅰ | G1 | T1BN0M0 | survival |
| 201512035 | 63 | F | ESCC | Ⅱ | G1 | T3N0M0 | survival |
| 201512176 | 67 | F | ESCC | Ⅱ | G1 | T2N0M0 | survival |
| 201512177 | 64 | M | ESCC | Ⅰ | G1 | T1BN0M0 | 2018/7/9 |
| 201512559 | 59 | M | ESCC | Ⅲ | G1 | T3N1M0 | 2017/9/20 |
| 201512600 | 74 | F | ESCC | Ⅲ | G1 | T3N1M0 | 2017/2/19 |
| 201512601 | 72 | M | ESCC | Ⅲ | G2 | T3N2M0 | 2016/3/23 |
| 201512602 | 57 | M | ESCC | Ⅲ | G1 | T3N2M0 | 2016/12/6 |
| 201513165 | 63 | M | ESCC | Ⅱ | G1 | T2N0M0 | survival |
| 201513229 | 80 | M | ESCC | Ⅲ | G3 | T3N1M0 | 2016/6/14 |
| 201513268 | 63 | F | ESCC | Ⅱ | G1 | T3N0M0 | 2016/6/13 |
| 201513471 | 61 | M | ESCC | Ⅲ | G2 | T3N1M0 | 2016/12/18 |
| 201513503 | 60 | M | ESCC | Ⅲ | G3 | T3N1M0 | 2018/9/14 |
| 201513624 | 63 | M | ESCC | Ⅱ | G1 | T2N0M0 | survival |
| 201503931 | 73 | M | ESCC | Ⅱ | G1 | T2N0M0 | survival |
| 201504023 | 58 | F | ESCC | Ⅲ | G3 | T3N2M0 | 2017/2/13 |
| 201504286 | 37 | M | ESCC | Ⅱ | G3 | T3N0M0 | 2017/6/13 |
| 201504289 | 59 | F | ESCC | Ⅱ | G3 | T2N0M0 | 2017/3/16 |
| 201504321 | 72 | M | ESCC | Ⅲ | G1 | T3N1M0 | 2016/8/13 |
| 201507433 | 59 | M | ESCC | Ⅰ | G1 | T1BN0M0 | 2018/11/4 |
| 201509634 | 52 | M | ESCC | Ⅱ | G1 | T3N0M0 | 2018/4/27 |
| 201509765 | 62 | F | ESCC | Ⅳ-B | G2 | T3N0M1 | 2016/10/31 |
| 201509766 | 52 | M | ESCC | Ⅲ | G3 | T3N1M0 | 2016/7/31 |
| 201511781 | 65 | M | ESCC | Ⅳ-A | G2 | T3N3M0 | 2017/6/14 |
| 201512030 | 64 | M | ESCC | Ⅱ | G2 | T3N0M0 | 2018/1/5 |
